# Effect of oat supplementation interventions on cardiovascular disease risk markers: a systematic review and meta-analysis of randomized controlled trials

**DOI:** 10.1007/s00394-021-02763-1

**Published:** 2022-01-03

**Authors:** Erand Llanaj, Gordana M. Dejanovic, Ezra Valido, Arjola Bano, Magda Gamba, Lum Kastrati, Beatrice Minder, Stevan Stojic, Trudy Voortman, Pedro Marques-Vidal, Jivko Stoyanov, Brandon Metzger, Marija Glisic, Hua Kern, Taulant Muka

**Affiliations:** 1 Department of Public Health and Epidemiology, Faculty of Medicine, University of Debrecen, Debrecen, Hungary; 2 Present Address: MTA-DE Public Health Research Group, Hungarian Academy of Sciences and University of Debrecen, Debrecen, Hungary; 3 Department of Ophthalmology, Faculty of Medicine, University of Novi Sad, Novi Sad, Serbia; 4 Swiss Paraplegic Research, Nottwil, Switzerland; 5 Department of Epidemiology, Erasmus MC University Medical Centre Rotterdam, Rotterdam, The Netherlands; 6 Division of Human Nutrition and Health, Wageningen University, Wageningen, The Netherlands; 7 Department of Medicine, Internal Medicine, Lausanne University Hospital, Lausanne, Switzerland; 8 University of Lausanne, Lausanne, Switzerland; 9 Institute of Social and Preventive Medicine (ISPM), University of Bern, Bern, Switzerland; 10 Faculty of Medicine, University of Prishtina “Hasan Prishtina”, Prishtina, Kosovo; 11 Public Health and Primary Care Library, University Library of Bern, University of Bern, Bern, Switzerland; 12 Standard Process Nutrition Innovation Centre, Kannapolis, USA; 13 Graduate School for Health Sciences, University of Bern, Bern, Switzerland; 14 Department of Cardiology, University Hospital of Bern, University of Bern, Bern, Switzerland

**Keywords:** Oats, Supplementation, Interventions, Cardiovascular diseases, Risk markers, Cholesterol, Nutrition

## Abstract

**Purpose:**

Oat supplementation interventions (OSIs) may have a beneficial effect on cardiovascular disease (CVD) risk. However, dietary background can modulate such effect. This systematic review assesses the effects of OSIs on CVD risk markers among adults, accounting for different dietary backgrounds or control arms.

**Methods:**

We included randomized clinical trials (RCTs) that assessed the effect of oat, oat beta-glucan-rich extracts or avenanthramides on CVD risk markers.

**Results:**

Seventy-four RCTs, including 4937 predominantly hypercholesterolemic, obese subjects, with mild metabolic disturbances, were included in the systematic review. Of these, 59 RCTs contributed to the meta-analyses. Subjects receiving an OSI, compared to control arms without oats, had improved levels of total cholesterol (TC) [weighted mean difference and (95% CI) − 0.42 mmol/L, (− 0.61; − 0.22)], LDL cholesterol [− 0.29 mmol/L, (− 0.37; − 0.20)], glucose [− 0.25 nmol/L, (− 0.36; − 0.14)], body mass index [− 0.13 kg/m^2^, (− 0.26; − 0.01)], weight [− 0.94 kg, (− 1.84: − 0.05)], and waist circumference [− 1.06 cm, (− 1.85; − 0.27)]. RCTs on inflammation and/or oxidative stress markers were scarce and with inconsistent findings. RCTs comparing an OSI to heterogeneous interventions (e.g., wheat, eggs, rice, etc.), showed lowered levels of glycated haemoglobin, diastolic blood pressure, HDL cholesterol and apolipoprotein B. The majority of included RCTs (81.1%) had some concerns for risk of bias.

**Conclusion:**

Dietary OSIs resulted in lowered levels of blood lipids and improvements in anthropometric parameters among participants with predominantly mild metabolic disturbances, regardless of dietary background or control. Further high-quality trials are warranted to establish the role of OSIs on blood pressure, glucose homeostasis and inflammation markers.

**Supplementary Information:**

The online version contains supplementary material available at 10.1007/s00394-021-02763-1.

## Introduction

Cardiovascular diseases (CVDs) represent one of the leading causes of global mortality among adults and lifestyle modifications have emerged as a great opportunity to reduce their health burden [1]. Hence, changes in diet have been encouraged, as they can have a beneficial impact on the prevention, management and disease trajectory of CVDs [2]. Among currently implemented dietary interventions, increased intake of whole grains and in particular oat components, such as oat fibre and oat bioactive constituents, has been suggested to affect CVD risk markers including blood cholesterol, blood glucose and body mass index (BMI), thus reducing the risk of coronary heart disease [3–6]. There is growing evidence suggesting that oat products, when compared with similar wheat-based products or a glucose control, can have a positive effect on human glycaemic response [7]. Similar positive effects have been also reported for overall CVD risk [8], satiety [9] and increased gut microbiota diversity [10]. Currently, a considerable number of randomized controlled trials (RCTs) and reviews have documented the health benefits that oat supplementation interventions (OSIs) confer, but such efforts are limited to a basic subset of CVD risk markers [6, 11]. In addition, little attention has been given to the role of background diet and control arm in the interpretation of the relationship between OSIs and CVD markers. Differentiating such effects [12, 13] by type of dietary OSI and/or control arm (e.g., oat-free intervention, low-fat diet, wheat, rice, etc.) can aid in harnessing the potential benefits of small, but consistent dietary changes, such as supplementing one’s diet with oats. With that in mind, we aimed at examining the effect of OSIs on a more extended set of CVD risk markers, while also taking into consideration dietary backgrounds and type of control arms used in the RCTs that explored how OSIs affected CVD risk markers. Following this rationale and based on the available RCTs, three major sub-classes emerged as follows: (i) RCTs comparing an OSI vs. oat-free diet or control product without oats, (ii) intervention group combining an OSI with some type of dietary restriction (e.g., low-fat diet, hypocaloric diet, etc.) vs. the same dietary restriction alone and (iii) an OSI vs. heterogeneous control arms (e.g., rice, eggs, fibre, wheat, etc.). Based on this categorization, we assessed the association of OSIs and CVD risk markers in adults, accounting for each subclass.

## Materials and methods

### Search strategy and study selection

This work follows an established guide on conducting systematic reviews and meta-analyses for medical research [14], as well as PRISMA [15] guidelines for reporting. An experienced medical librarian systematically searched four electronic databases: EMBASE, MEDLINE (Ovid), Cochrane Central and Web of Science from inception until May 15, 2020 (date last searched); additionally, the first 200 results were downloaded from the Google Scholar search engine. A detailed search strategy is outlined in the supplementary material (section Search strategy). We additionally performed a hand search of the reference lists of included RCTs. Detailed inclusion and exclusion criteria can be found in the review protocol PROSPERO (ID: CRD42020189278). In brief, RCTs were included only if they (i) were conducted in humans and (ii) investigated the associations of oat, oat beta-glucan-rich extracts (OβGREs) and/or avenanthramides dietary supplementation with any of the following outcomes: serum lipid profile, glucose homeostasis parameters, inflammatory and oxidative stress markers, body morphology parameters and/or blood pressure.

### Data extraction and assessment of the quality of included studies

Two reviewers, who afterwards assessed the full-texts of potentially eligible studies, independently evaluated titles and abstracts. Two reviewers also independently extracted the relevant information using a pre-defined data extraction form. Any disagreement between reviewers was settled by reaching a consensus or by consulting a third reviewer. The quality of included RCTs was assessed by two independent reviewers using the Risk of Bias Tool for Randomized Trials (Rob 2.0) [16]. Detailed information on the assessment of study quality and risk of bias is provided in Table 1.

**Table 1:** Study characteristics of the RCTs included in the systematic review

Ref	Lead Author (publication year)	Location	RCT design	Sample size, (*n*)	Male participants, *n* (%)	Health status of study sample	Mean age, in years (SD)*	Duration	Characteristics of intervention arm	Characteristics of control arm	Overall risk of bias	Isocaloric diet	Different intake or background diet between arms
[56]	Abrahamsson et al. (1994)	Sweden	C	31	0 (0)	Healthy female subjects	26 (6.5)	Two 5-week periods	Oat bran	Wheat bran	SC	–	No
[57]	Adamsson et al. (2015)	Sweden	P	79	31 (39.2)	Mildly hypercholesterolemic and overweight subjects	54.6 (10.8)	12 weeks	Oat bran (porridge or muesli)—40 g per serving (corresponding to 3 g/d oat β-glucans)	Usual breakfast	SC	–	No
[58]	Amundsen ÅL et al. (2003)	Sweden	C	16	9 (56.3)	Hypercholesterolaemic subjects	57 (7.9)	Two 3-week periods	OβGRE corresponding to 5 g/d oat β -glucans	Diet without OβGREs	SC	Yes	No
[59]	Anderson et al. (1990)	USA	P	14	14 (100)	Hypercholesterolemic subjects	58	2 weeks	Oat bran (56 g/d)	Corn flakes (56 g/d)	H	Yes	No
[60]	Anderson et al. (1991)	USA	P	21	21 (100)	Hypercholesterolemic male subjects	61 (2)	3 weeks	Oat bran as cereal and muffins (110 g/d)	Wheat bran (40 g/d)	SC	Yes	No
[61]	Anderson et al. (1984)	USA	P	20	20 (100)	Hypercholesterolemic male subjects	Range (34–66)	3 weeks	Oat-bran – 100 g/d of oat bran (dry wt.) served as cereals and oat bran muffins	Beans – diet containing 115 g of dried bean (dry wt.)	SC	Yes	No
[62]	Ballesteros et al. (2015)	Mexico	C	29	10 (34.5)	Subjects with type 2 diabetes	53.5 (8.3)	2 periods of 5 weeks	40 g/d of oatmeal with 2 cups (472 mL) of lactose-free milk	One egg daily	SC	Yes	No
[63]	Beck et al. (2010)	Australia	P	56	0 (0)	Overweight female subjects	37.4 (5.3)	12 weeks	2 MJ energy-deficit diets with high-fibre products with added OβGREs providing β-glucans at a moderate (5–6 g/d) and at a high (8–9 g/d) level	2 MJ energy-deficit diets with high-fibre products and no oat OβGREs	L	Yes	No
[64]	Berg et al. (2003)	Germany	P	288	288 (100)	Male subjects with increased risk for coronary heart disease	53.6 (6.3)	4 weeks	Group 1: fat-modified diet (NCEP step 2) with caloric restriction to 1,000 kcal/d and in addition a daily intake of 35–50 g of oat bran Group 2: fat-modified diet (NCEP step 2) with caloric restriction to 1,000 kcal/d	Control group: age- and weight-matched normocholesterolemic overweight males; 1,000 kcal/d and only moderately fat-modified diet (NCEP step 1)	SC	No	Yes
[65]	Biörklund et al. (2005)	Sweden	P	89	44 (49.4)	Hypercholesteraemic subjects	18–70	5 weeks	Beverage with 5 or 10 g β-glucans extract from oats or barley	Control beverage enriched with rice starch	H	No	Yes
[66]	Biörklund et al. (2008)	Sweden	P	43	19 (44.2)	Hyperlipidaemic subjects	58 (8.2)	8 weeks	Soup with OβGREs, providing 4 g/d oat β-glucans	Soup without OβGREs	SC	Yes	No
[67]	Braaten et al. (1994)	USA	C	30	N.R	Hypercholesterolemic subjects	N.R	Two periods of 4 weeks	Oat gum with 2.9 g of β-glucans	Placebo (maltodextrin)	H	Yes	No
[68]	Bremer et al. (1991)	New Zealand	C	12	5 (41.7)	Hyperlipidaemic subjects	53 (10)	12 weeks	Oat bread—six slices daily for females and 10–12 slices daily for males	Wheat bread—six slices daily for females and 10–12 slices daily for males	SC	Yes	No
[69]	Bridges et al. (1992)	USA	P	20	20 (100)	Hypercholesterolemic male subjects	61 (range 38–73)	3 weeks	Oat bran 110 g/d (dry wt.), served as a bowl of hot cereal and oat-bran muffins	Wheat-bran diets provided 40 g/d wheat bran (dry wt.) served as a bowl of ready-to-eat cereal and wheat-bran muffins	SC	Yes	No
[70]	Chang et al. (2013)	USA	P	34	12 (35.3)	Overweight and obese subjects	38.5 (11.3)	12 weeks	β-glucans -containing cereal. One cereal pack (37.5 g) was prescribed to be mixed with 250 mL hot water twice daily	Placebo (cereal without β-glucans)	SC	No	Yes
[71]	Chen et al. (2006)	USA	P	102	41 (40)	Healthy subjects	47.9 (8.4)	12 weeks	60 g of oat bran concentrate as a muffin and 84 g of oatmeal squares	93 g of refined wheat as a muffin and 42 g of corn flakes	L	No	Yes
[72]	Connolly et al. (2016)	England	C	30	11 (36.7)	Subjects with glucose intolerance or mild to moderate hypercholesterolemia	42 (N.R.)	Two 6-week periods	Whole grain oat granola cereal (45 g/d)	Non-whole grain breakfast; 45 g/d	SC	Yes	No
[73]	Davy et al. (2002)	USA	P	36	36 (100)	Overweight male subjects	58 (8.6)	12 weeks	60 g oatmeal and 76 g oat bran ready-to-eat cold cereal and the wheat group consumed 5.5 g β-glucans	60 g whole wheat cereals and 81 g frosted mini-wheats	SC	No	Yes
[74]	De Souza et al. (2016)	Brazil	P	132	44 (33.3)	Hypercholesterolemic subjects	55.8 (10.6)	~ 13 weeks	40 g of oat bran	40 g of corn starch and rice flour	SC	No	Yes
[75]	Dubois et al. (1993)	France	P	6	6 (100)	Normolipidemic male subjects	Range (20–27)	2 weeks	Usual low-fibre diet and oat bran (40 g/d)	Usual low-fibre diet	SC	No	Yes
[76]	Ferguson et al. (2020)	Australia	P	72	27 (37.5)	Hypercholesterolemic subjects	55.1 (1.4)	6 weeks	Biscuits fortified with 2 g phytosterols (Group 1), 3 g β-glucans (Group 2) and 2 g phytosterols and 3 g β-glucans (Group 3)	Placebo (biscuit without phytosterols and β-glucans)	SC	Yes	No
[77]	Geliebter et al., 2014	USA	P	36	18 (50)	Overweight subjects	33.9 (7.5)	4 weeks	Oat porridge or frosted cornflakes	No-breakfast	SC	Yes	No
[78]	Gerhardt et al. (1998)	USA	P	44	23 (52.3)	Moderately hypercholesterolemic subjects	51.7 (1.5)	6 weeks	Low-fat diet and oat bran; 84 g/d	Low-fat diet and rice starch placebo; 84 g/d	SC	Yes	No
[79]	Guevara-Cruz et al. (2012)	Mexico	P	67	N.R	Subjects with metabolic syndrome	Range (20–60)	8 weeks	Habitual diet reduced by 500 kcal and 22 g oats	Placebo: habitual diet reduced by 500	H	No	Yes
[80]	Gulati et al. (2017)	India	P	69	N.R	Mildly hypercholesterolemic subjects	31.2 (6.6)	4 weeks	35 g of oats twice daily (total of 70 g/d) in the form of porridge (35 g of oats) for breakfast and a second serving of oats in the form of Upma (35 g of oats) in the afternoon	Usual diet	SC	No	Yes
[81]	He et al. (2004)	USA	P	102	N.R	Subjects with stage 1 hypertension or increased blood pressure	47.7 (8.5)	12 weeks	High fibre: group received a daily serving of 60 g oat bran concentrate as a muffin and 84 g oatmeal squares	Low fibre: 93 g of refined wheat as a muffin and 42 g corn flakes	L	No	Yes
[82]	Hegsted et al. (1993)	USA	C	11	10 (90.9)	Mildly hypercholesterolemic subjects	37 (33.2)	Two periods of 3 weeks	100 g/d oat bran	100 g/d stabilized rice bran	SC	Yes	No
[83]	Ibrugger et al. (2013)	Denmark	C	14	6 (42.6)	Healthy subjects	22.9 (2.1)	Four 3-week periods	Beverage of 3.3 g/d oat, barley, and barley mutant b-glucans’ extract of similar molecular mass	Control beverage	SC	Yes	No
[84]	Johansson-Persson et al. (2014)	Sweden	C	30	12 (34.3)	Healthy subjects	58.6 (1.1)	Two 5-week periods	Oat bran beverage combined with a high-fibre diet, providing 4.4 g total dietary fibre per day (corresponding to 2.8 g β-glucans)	The rice beverage in the low-fibre diet provided 0.4 g fibre daily	SC	Yes	No
[85]	Kabir et al. (2002)	France	C	13	13 (100)	Subjects with type 2 diabetes	59 (7.2)	Two 4-week periods	Low-glycaemic index breakfast period, the cereal used was based on extruded oat bran concentrate, apple, and fructose (muesli containing 3 g β-glucans). The bread used was pumpernickel	High-glycaemic index breakfast whole wheat grains and whole meal bread (wheat flour)	SC	Yes	No
[86]	Karmally et al. (2005)	USA	P	152	49 (32.2)	Healthy subjects	49 (10.6)	11 weeks	Ready-to-eat oat cereal (portion size: 45 g/d)	Corn Cereal	H	No	-
[87]	Kashtan et al. (1992)	Canada	P	84	50 (59.5)	Subjects with a history of previous polypectomy and volunteers with normal colon on colonic examination	55.8 (13)	2 weeks	Oat bran twice per day (88.4 g/d)	Wheat bran twice per day (73 g/d)	SC	Yes	No
[88]	Keenan et al. (1991)	USA	C	75	49 (65.3)	Healthy subjects	Range (20–70)	Three periods of 6 weeks	AHA Step I diet and oat bran, 28 g/d	AHA Step I diet and wheat bran	H	Yes	No
[89]	Keenan et al. (2002)	USA	P	18	N.R	Hypertensive and hyperinsulinemic subjects	44 (18)	6 weeks	Oat cereals providing ~ 5.5 g/d of β-glucans	Low-fibre cereal (< 1 g/d total fibre)	SC	Yes	No
[90]	Kerckhoffs et al. (2003)	The Netherlands	P	48	21 (43.8)	Healthy subjects	53 (13.9)	4 weeks	Bread and cookies rich in β-glucans (~ 1.5 g/d) from > 5 g/d oat bran	bread and cookies rich in wheat fibre	SC	No	Yes
[91]	Kirby et al. (1981)	USA	P	8	8 (100)	Hypercholesterolemic subjects	Range (35–62)	2 weeks	Diet containing 100 g of oat-bran daily, provided in form of muffins and cereals	Diet composed of commonly available foods	SC	Yes	No
[92]	Kristensen et al. (2011)	Denmark	C	24	N.R	Healthy subjects	25.2 (2.7)	Two periods of 2 weeks	Low-fibre diet and 102 g/d oat bran	Low-fibre diet	SC	Yes	No
[93]	Laaksonen et al. (2005)	Finland	P	72	36 (50)	Subjects with metabolic syndrome	55.4 (6.8)	12 weeks	Oat bread (made of 60% whole meal oat flour and 40% wheat flour)	Rye-pasta	SC	Yes	No
[94]	Leadbetter et al. (1991)	USA	P	40	20 (50)	Hypercholesterolemic subjects	Range (25–64)	4 months	30, 60 or 90 g/d oat bran	No supplementation	SC	Yes	No
[37]	Leão et al. (2019)	Brazil	P	154	41 (26.6)	Subjects with metabolic syndrome	47.6 (12.6)	6 weeks	Low-calorie diet plus oat bran (40 g/d)	A low-calorie diet	SC	No	Yes
[10]	Li et al. (2016)	China	P	298	155 (52)	Overweight subjects with type 2 diabetes	59.5 (6)	4 weeks	Diet with the same quantity of cereals replaced by 50 g and 100 g oats respectively	Low-fat and high-fibre diet	SC	No	-
[95]	Liao et al. (2019)	Taiwan	P	74	N.R	Healthy and mildly hypercholesterolemic subjects	Range (35–70)	10 weeks	Oat noodles containing 12 g of β-glucans	Wheat noodles	SC	No	Yes
[96]	Liatis et al. (2009)	Greece	P	41	23 (56.1)	Subjects with type 2 diabetes	62.9 (9.1)	3 weeks	Bread enriched β-glucans (providing 3 g/d β-glucan)	Bread without β-glucans	H	No	Yes
[97]	Liu et al. (2011)	China	P	120	60 (50)	Healthy subjects	N.R	4 weeks	Either 4 capsules containing 1.6 mg of oat avenanthramides or 8 capsules containing oat avenanthramides-enriched extract (3.1 mg)	Placebo capsules (corn oil) or no treatment at all (control group)	H	No	No
[98]	Lovegrove et al. (2000)	UK	P	62	31 (50)	Healthy subjects	56.6 (9.4)	8 weeks	20 g oat bran concentrate providing 3 g β-glucans	20 g wheat bran	SC	No	No
[99]	Maki et al. (2003)	USA	P	112	49 (43.8)	Hypercholesterolemic subjects	57.3 (9.5)	6 weeks	Cereal, a snack bar and a beverage with 1.8 g oil–based phytosterols and 2.8 g/d β-glucans	Cereals, a snack bar and a beverage with less than 1 g β-glucans daily, and no oil–based phytosterols	SC	No	Yes
[100]	Maki et al., (2007)	USA	P	60	33 (55)	Subjects with elevated blood pressure	59.7 (9.4)	12 weeks	A ready-to-eat cold cereal made with oat bran, oatmeal and a powdered form of β-glucans	(1) A low-fibre ready-to-eat cold wheat-based cereal (2) a low-fibre hot cereal and (3) a control maltodextrin powder	SC	No	Yes
[101]	Maki et al. (2010)	USA	P	144	31 (21.5)	Healthy subjects	48.9 (10.2)	12 weeks	Energy deficit of 500 kcal/d and wholegrain oat cereals containing ~ 3 g/d β-glucans	Energy deficit of 500 kcal/d and low-fibre breakfast/snack foods	SC	No	Yes
[102]	Martensson et al. (2005)	Sweden	P	56	24 (42.9)	Moderately hypercholesterolemic subjects	55 (9)	3 weeks run-in, 5 weeks intervention	Fermented oat-based product (3–3.5 g/d native β-glucans) and oat-based product ropy which was co-fermented with an exopolysaccharide-producing strain (*Pediococcus damnosus)*	Fermented dairy-based product	SC	No	–
[103]	Missimer et al. (2017)	USA	C	50	24 (48)	Healthy subjects	23.3 (3.1)	Two periods of 4 weeks	Oatmeal 35 g/d for breakfast	2 eggs for breakfast, daily	SC	Yes	No
[104]	Momenizadeh et al. (2014)	Iran	P	60	21 (35)	Hypercholesterolemic subjects	51.1 (9.3)	6 weeks	Five servings of oat bread providing 6 g β-glucans	At least five servings of wheat bread	SC	No	Yes
[105]	Noakes et al. (1996)	Australia	C	23	13 (56.5)	Overweight, obese, dyslipidemic and/or hypertensive subjects	51 (6.7)	Three periods of 4 weeks	Oat bran	Two control diets: high-amylose and low-amylose diet	SC	Yes	No
[106]	Önning et al. (1999)	Sweden	C	66	66 (100)	Moderate hypercholesterolemia	Mean age (62.6); Range (52–70)	Two periods of 5 weeks	Oat milk (0.75 L, daily)	Rice milk (0.75 L, daily)	SC	Yes	No
[107]	Önning et al. (1998)	Sweden	P	11	6 (54.5)	Healthy, non-smoking subjects	Range (23–54)	4 weeks	Oat milk daily (0.75 L for females and 1 L for males)	Cow’s milk was a medium-fat milk (0.75 L for females and 1 L for males daily)	SC	Yes	No
[20]	Pavadhgul et al. (2019)	Thailand	C	24	N.R	Hypercholesterolemic subjects	Range (30–60)	Two 4-week periods	70 g of instant oat flakes (porridge)	70 g instant white rice flakes (porridge)	SC	Yes	No
[108]	Pins et al. (2002)	USA	P	88	45(51.1)	Subjects with history of essential mild or moderate hypertension	47.6 (16.1)	Three 4-week periods	60 g Oatmeal and 77 g Oat Squares	65 g wheat cereals and 81 g of rice- and corn-based breakfast cereals	SC	No	-
[109]	Poulter et al. (1994)	UK	C	59	17 (28.8)	Hypercholesterolemic subjects	56.3 (2.5)	2 periods of 4 weeks	Oat-based cereal (50 g)	Usual cereal without oat	SC	Yes	No
[22]	Queenan et al. (2007)	USA	P	75	25 (33.3)	Hypercholesterolemic subjects	44.9 (12.9)	6 weeks	6 g/d concentrated β-glucans (powder form)	6 g/d dextrose monohydrate (powder)	H	-	No
[110]	Reyna-Villasmil et al. (2007)	Venezuela	P	38	38 (100)	Mild to moderate hypercholesterolemic subjects	59.8 (0.6)	8 weeks	AHA Step II diet plus bread containing 6 g/d of oat-derived β-glucans	Same diet as the intervention arm plus whole-wheat bread providing 6 g/d of fibre	SC	Yes	No
[111]	Robitaille et al. (2005)	Canada	P	34	0 (0)	Normal cycling premenopausal overweight female subjects	38.3 (7.5)	4 weeks trial (2-week run-in phase)	28 g/d of oat bran in form of oat bran-enriched muffins	No supplement	SC	No	No
[112]	Romero et al. (1998)	Mexico	P	46	46 (100)	Sedentary hypercholesterolemic male subjects	Range (20–45)	8 weeks	Oat bran–100 g of cookies daily which is equivalent to 2.8 g of soluble fibre derived from oat bran	Wheat bran–100 g of cookies daily which is equivalent to 0.6 g of soluble fibre derived from wheat bran	SC	Yes	No
[113]	Saltzman et al. (2001)	USA	P	43	20 (46.5)	Healthy subjects	44.6 (27.5)	6 weeks	Hypocaloric diet and oats – 45 g/ (4.2 MJ dietary energy daily)	Hypocaloric diet without oat	SC	No	Yes
[114]	Schweinlin et al. (2018)	Germany	P	36	13 (36.1)	Obese subjects with NAFLD	49.9 (10.3)	2 + 10 weeks intervention	Powdered diet supplement containing 6 g oatmeal, enriched with 1,7 g β-glucans and 5 g oat fibre–3 × 30 g/d (2 weeks) and 2 × 30 g/d (10 weeks)	Low-glycaemic and insulinemic diet	SC	No	Yes
[115]	Tabesh et al. (2014)	Iran	P	60	21(35)	Hypercholesterolemic subjects	51.1 (9.3)	4 weeks	Hypocaloric diet with 150 g oat bread rich in β-glucan–corresponding to 18 g/d of β-glucans	Hypocaloric diet with 150 g wheat bread rich in wheat fibre, but no β-glucan	SC	No	Yes
[116]	Theuwissen et al. (2009)	The Netherlands	C	42	20 (47.6)	Healthy subjects	52 (11)	2 periods of 4 weeks	β-glucan -containing muesli (4.8 g β-glucan)	Muesli without with 4.8 g fibre	SC	Yes	No
[117]	Thongoun et al. (2013)	Thailand	C	24	2 (8.3)	Hypercholesterolemic subjects	51 (6.9)	2 periods of 4 weeks	Oat bran 70 g (corresponding to 3 g β-gluans)	70 g rice porridge	SC	Yes	No
[23]	Tighe et al. (2010)	UK	P	206	105 (51)	Healthy subjects	51.8 (7.4)	12 weeks intervention	35–40 g whole meal bread plus 60–80 g of whole grain rolled oats daily	70–80 g whole meal bread plus 30–40 g whole grain cereals or 3 servings of refined cereals foods, daily	SC	No	Yes
[118]	Trinidad et al. (2004)	Philippines	C	21	4 (19)	Mildly hypercholesterolemic subjects	48.4 (4.6)	Four 2-week periods, separated by 2 weeks washout	50 g organic oat bran flakes daily	3 comparisons: 50 g corn flakes; 50 g cornflakes with 15% coconut flakes; 50 g 25% coconut flakes	SC	Yes	No
[120]	Uusitupa et al. (1992)	Finland	P	36	20 (55.6)	Hypercholesterolemic subjects	47.8 (7.6)	8 weeks	29.8 g oat bran (corresponding to 10.3 g/d β-glucans)	20.5 g/d wheat bran	SC	-	No
[119]	Uusitupa et al. (1997)	Finland	P	36	20 (55.6)	Hypercholesterolemic subjects	47.8 (7.6)	8 weeks	29.8 g oat bran (corresponding to 10.3 g/d β-glucans)	20.5 g/d wheat bran	H	-	No
[121]	Van Horn L et al. (1991)	USA	P	80	40(50)	Hypercholesterolemic subjects	42.5 (12.9)	8 weeks	Two packets (56.7 g/d dry wt.) of instant oats	Usual intake	SC	No	-
[122]	Vuksan et al. (2017)	Canada	P	58	18 (31)	Overweight and obese subjects with type 2 diabetes	60 (2)	26 weeks (6 months)	25.7 g/d oat bran	30 g/1000 kcal of ground Salba-chia	SC	No	Yes
[123]	Wolever et al. (2010)	Canada	P	367	210 (57.2)	Healthy subjects	53.5 (9.1)	4 weeks	Oat bran containing 3–4 g/d β-glucans	Wheat bran	L	No	Yes
[124]	Zhang et al. (2012)	China	P	166	65 (39.2)	Subjects with mild to moderate hypercholesterolemia	53.2 (6.5)	6 weeks	100 g/d of instant oat cereal	100 g/d of wheat flour-based noodles	SC	No	Yes

*N.R*. value not reported or could not be found**;** *OβGREs* oat beta-glucan-rich extracts; *kcal*/*d* kilocalories per day; *g*/*d* grams per day *NCEP* National Cholesterol Education Program; *AHA* American Heart Association; *dry wt*. dry weight *NAFLD* Non-alcoholic fatty liver disease; *C* cross-over RCT design; *P* parallel RCT design; *H* High risk of bias; *L* Low risk of bias; *SC* some concerns for bias

*Values are given as mean and (standard deviation) unless otherwise indicated

### Statistical analysis

Treatment effects were defined as the pre-post differences in outcomes between OSIs and control arms at the end of a RCT. All outcomes were continuous, therefore, the mean differences [intervention minus control] of the treatment effects in CVD risk markers were presented as summary outcome measures. For data reported as medians, ranges or 95% confidence intervals (CI), means and standard deviations were converted as described elsewhere [17]. Random-effect models were used to obtain estimates of weighted mean differences (WMDs) and 95%CIs. For RCTs with crossover design, we used the data from the first study period only. Due to observed variations between the definition of intervention and control diet across different RCTs, we pooled the effect estimates by grouping the following type of RCTs based on background diet and control arm: (i) an OSI group compared with the same/other intervention groups, but without oats (ii) intervention group combining an OSI and some type of dietary restriction (DR) (e.g., low-fat diet, hypocaloric diet, etc.) versus the same DR without oats, and (iii) an OSI compared with other interventions (e.g. rice, eggs, fibre, wheat, etc.). Henceforth these groups will be referred to their short form as (i) OSI vs. no OSI controls, (ii) OSI + DR vs. DR alone and (iii) OSI vs. heterogeneous controls, respectively. Heterogeneity between studies was assessed using the Cochrane *χ*^2^ statistic (P_q_ < 0.05 was considered as significant) and the *I*^*2*^ statistic, and was determined as low (*I*^*2*^ ≤ 25%), moderate (25% < *I*^*2*^ < 75%), or high (*I*^*2*^ ≥ 75%) [18]. Study characteristics including geographic location, number of participants, duration of intervention, baseline age, health status (healthy individuals vs. those with pre-existing health conditions), and study quality were pre-specified as characteristics for assessment of heterogeneity, and were evaluated using stratified analyses and random-effects meta-regression, if eight or more studies were included in the meta-analysis [19]. We performed a leave-one-out sensitivity analysis iteratively by removing one study at a time to explore whether any single study influenced the results. Publication bias was evaluated through visual inspection of funnel plot and Egger’s test. All statistical analyses were conducted with STATA, Release 16 (Stata Corp, College Station, Texas, USA). The RCTs that could not be quantitatively pooled were qualitatively summarized.

## Results

### Included studies

**Fig. 1:**
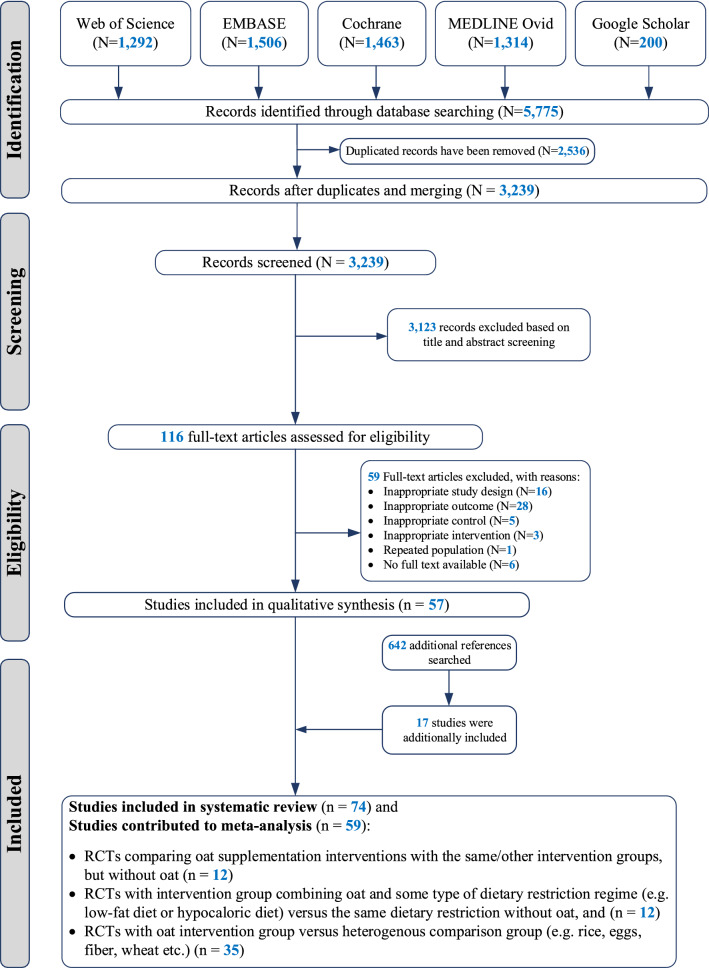
PRISMA flowchart of selection process and included studies

Of 3239 unique citations yielded from the search strategy, 116 relevant full-text articles were retrieved, of which 57 RCTs met all eligibility criteria. We screened the reference lists of those 57 RCTs and identified an additional 17 studies that met all criteria. As a result, a total of 74 RCTs were included in the systematic review, comprising 4,937 individuals. Among the 74 included RCTs, only 59 could be included in the meta-analysis (Fig. 1). Twenty-nine RCTs were conducted in North America, twenty-five in Europe, thirteen in Asia–Pacific and seven in South America. The sample size ranged from 6 to 298 individuals (median 45, interquartile range (IQR): 36–60) and the duration of the interventions from 2 to 26 weeks (median 8 weeks, IQR: 4.25–12). The majority of RCTs (*n* = 56, 75.7%) included individuals with some form of metabolic disturbance (i.e., type 2 diabetes (T2D), metabolic syndrome, prediabetes, prehypertension, hyperlipidaemia), while only 18 RCTs were conducted in healthy individuals. The majority of the RCTs (*n* = 60, 81.1%) investigated oat bran, meal or porridge supplementation, 13 RCTs reported on β-glucan- containing oat products and one investigated avenanthramides (Table 1). Only 35 (47.3%) out of 74 RCTs took energy intake into account between trial arms. The majority of studies (60 out of 74, 81.1%) were evaluated as having some concerns about risk of bias, mostly due to issues linked to randomization, allocation and blinding. Ten studies (13.5%) had high risk of bias and four studies (5.4%) had low risk of bias (see Tables 1 and 2). Among the 59 RCTs included in the meta-analysis, 12 contributed to the main meta-analysis (comparing OSI vs. no OSI controls), 12 contributed to the meta-analysis comparing an OSI + DR vs. DR alone, and 35 contributed to the meta-analysis comparing an OSI vs. heterogeneous control arms.

**Table 2:** Meta-analysis of randomized clinical trials comparing oat supplementation interventions with diet or control product without oats

	Included studies		Participants			Study quality				Meta-analysis results		
Outcome	No. of unique studies	Follow-up duration, median (IQR), weeks	Total, no	Median sample size per intervention arm (IQR)	Age, median (IQR), years	No. of studies including healthy individuals, no. (%)	H	SC	L	WMD (95% CI)	*I*^2^ (%)	*P* value for heterogeneity
Body morphology												
BMI, kg/m^2^	5	5 (3.5;9)	249	43 (37.5;65.5)	42 (34.9;60.5)	0 (0)	1	4	0	**− 0.329 (− 0.634; − 0.025)**	55.6	0.060
Body weight, kg	5	4 (3;12)	250	41 (31;73.5)	38.5 (27.1;58.8)	1 (20)	1	4	0	**− 0.943 (− 1.842; − 0.045)**	52.3	0.090
Waist circumference, cm	3	4 (n.a.)	144	–	–	0 (0)	1	2	0	**− 1.058 (− 1.845; − 0.270)**	0.0	0.610
Body fat, %	1	–	–	–	–		–	–	–	–	–	–
Blood lipids												
Total cholesterol, mmol/L	12	4.5 (3.3;7.5)	589	38.5 (32.5;67.3)	44.5(36.5;57.3)	1 (8.3)	1	11	0	**− 0.415 (− 0.607; − 0.223)**	96.1	< 0.001
LDL, mmol/L	12	4.5 (3.3;7.5)	589	38.5 (32.5;67.3)	44.5 (36.5;57.3)	1 (8.)	1	11	0	**− 0.286 (− 0.372; − 0.200)**	72.6	< 0.001
HDL, mmol/L	12	4.5 (3.3;7.5)	589	38.5 (32.5;67.3)	44.5(36.5;57.3)	1 (8.3)	1	11	0	− 0.015 (− 0.041; 0.012)	46.5	0.030
Triglycerides, mmol/L	10	4.5 (3;9)	466	42 (33.5;78.5)	48.6(33.2;57.8)	1 (10)	1	9	0	− 0.022 (− 0.096; 0.052)	59.6	0.008
Glucose homeostasis												
Glucose, mmol/L	3	6 (n.a.)	146	–	–	0 (0)	1	2	0	**− 0.247 (− 0.357; − 0.136)**	47.29	0.150
HbA1c, %	0	–	–	–	–		–	–	–	–	–	–
Insulin, pmol/L	2	–		–	–	0 (0)	1	1	0	− 22.33 (− 49.66; 4.95)	66.0	0.090
Blood pressure												
Systolic blood pressure, mmHg	5	8 (3.5;12)	302	69 (37.5;79)	–	0 (0)	1	4	0	− 0.56 (− 1.68; 0.56)	33.8	0.200
Diastolic blood pressure, mmHg	5	8 (3.5;12)	302	69 (37.5;79)	–	0 (0)	1	4	0	− 0.69 (− 1.59; 0.22)	42.8	0.140

Significant weighted mean differences are bolded; *BMI* body mass index; *HbA1c* Glycated haemoglobin; *IQR* interquartile range; *WMD* Weighted mean difference; *I*^2^ variation across studies that is due to heterogeneity rather than chance; *n.a*. not available; *H* High risk of bias; *L* Low risk of bias; *SC* some concerns for bias

### Meta-analysis of RCTs comparing oat supplementation interventions with the same intervention without oat product

Twelve RCTs contributed to the main meta-analysis comparing the effects of an OSI vs. no OSI controls, on CVD risk markers. In this comparison, the OSI was associated with a higher decrease in total cholesterol (TC) [WMD and (95% CI) − 0.42 mmol/L, (− 0.61; − 0.22), *I*^2^ = 96.1%, *P*_q_ < 0.001] and low-density lipoprotein cholesterol (LDL-C) [− 0.29 mmol/L, (− 0.37; − 0.20), *I*^2^ = 72.6%, *P*_q_ < 0.001] (Table 2). In addition, glucose [− 0.25 mmol/L, (− 0.36; -0.14), *I*^2^ = 47.9%, *P*_q_ = 0.15], BMI [− 0.33 kg/m^2^, (− 0.63; − 0.03), *I*^2^ = 55.6%, *P*_q_ = 0.09], body weight [− 0.94 kg, (− 1.84: − 0.05) *I*^2^ = 52.3%, *P*_q_ = 0.09] and waist circumference (WC) [− 1.06 cm, (− 1.85; − 0.27), *I*^2^ = 0%, *P*_q_ = 0.61] were lower in the OSI group compared to the control arm (Table 2). We found no differences in high-density lipoprotein cholesterol (HDL-C), triglycerides (TGs), or blood pressure (BP) when comparing the OSI arm to that of no OSI controls (Table 2).

#### Subgroup analysis, leave-one-out analysis and publication bias

Subgroup analyses, meta-regression and analysis of sources of heterogeneity were conducted only if at least 8 studies were available. We identified high heterogeneity across different studies (*I*^*2*^ ranged from 0 to 96.1%). Due to the limited number of studies included in our analyses, we were able to explore sources of heterogeneity only in the meta-analysis of blood lipids (subgroup analyses were not performed if less than 8 studies contributed to meta-analyses). Besides the percentage of male study participants, which was identified as potential source of heterogeneity in case of LDL-C, the heterogeneity in the other meta-analyses of blood lipids was not explained by any individual participant nor study characteristics (e.g., age, health status, number of participants, duration of intervention and location) (Supplemental Table 2**)**. The findings were also supported by regressing continuous variables, such as age, duration of study and number of study participants—showing no evidence for linear association between those variables and WMD of TC, HDL-C, LDL-C and TGs (Supplemental Figs. 8–11). Due to the limited number of studies included, we could not stratify the meta-analyses based on intervention type (oat or OβGREs) or on intervention’s daily dose. The leave-one-out analyses did not show any study to influence the results on TC, HDL-C, LDL-C and TGs (Supplemental Tables 3–6); the leave-one-out analysis was not feasible for other outcomes due to the limited number of studies. We found no evidence for publication bias of RCTs included in meta-analysis comprising five or more estimates; funnel plots were in general symmetrical and Egger’s *p* values were higher than 0.05 (Supplemental Figs. 9–16).

### Meta-analysis of RCTs comparing oat supplementation intervention combined with some type of dietary restriction versus the same dietary restriction alone

Data from 12 RCTs were used to compare changes in CVD risk markers between intervention groups combining an OSI with some type of DR versus DR alone. When pooling the estimates of these RCTs, we found that: (i) BMI [WMD: − 0.13 kg/m^2^, 95% CI (− 0.26; − 0.02), *I*^2^ = 40%, Q_p_ = 0.13], TC [WMD: − 0.43 mmol/L, 95% CI (− 0.56; − 0.30), *I*^2^ = 91.7%, Q_p_ < 0.001], HDL-C [WMD: − 0.05 mmol/L, 95% CI (− 0.10; − 0.006), *I*^2^ = 97.1%, Q_p_ < 0.001], and LDL-C [WMD: − 0.26 mmol/L, 95% CI (− 0.38; − 0.14), *I*^2^ = 94.1%, Q_p_ < 0.001] were lower in the OSI + DR group compared to DR alone arm (Table 3). No differences were seen in apolipoproteins A and B between the two groups. In addition, HbA1c and diastolic BP were lower in OSI + DR group compared to DR alone, with WMD of − 0.42 mmol/L [(− 0.48; − 0.36), I^2^ = 0%, Q_p_ = 0.76] and− 1.15 mmHg [(− 2.03; − 0.28), *I*^2^ = 55.9%, Q_p_ = 0.06], respectively (Table 3).

**Table 3:** Meta-analysis of randomized clinical trials comparing oat supplementation combined with some type of dietary restriction versus the same dietary restriction alone

	**Included studies**		**Participants**			**Study quality**				**Meta-analysis results**		
Outcome	No. of unique studies	Follow-up duration, median (IQR), weeks	Total, no	Median sample size per intervention arm (IQR)	Age, median (IQR), years	No. of studies including healthy individuals, no. (%)	H	SC	L	WMD (95% CI)	I^2^ (%)	P value for heterogeneity
Body morphology												
BMI, kg/m^2^	6	4 (4–6.5)	788	62 (30–79)	59.5 (52.1–59.6)	0 (0)	1	6	0	− **0.129 (**− **0.256; **− **0.002)**	40.0	0.130
Body weight, kg	7	6 (4–10)	739	24 (19–79)	46.1 (37.4–59.5)	2 (28.6)	1	4	2	− 0.171 (− 0.486; 0.143)	39.2	0.110
Waist circumference, cm	6	7 (4–11.5)	706	31 (19–79)	48.7 (37.4–59.5)	0 (0)	1	5	0	0.146 (− 0.438; 0.730)	77.9	< 0.001
Body fat, %	2	–	384	–	–	(0)	0	2	0	0.316 (− 0.069; 0.701)	69.8	0.040
Blood lipids												
Total cholesterol, mmol/L	8	6 (4–9)	745	21 (16–72.5)	55.3 (39.2–59.5)	1 (12.5)	1	6	1	− **0.430 (**− **0.556; -0.304)**	91.7	< 0.001
LDL, mmol/L	9	6 (4–10)	804	22 (18.5–59.5)	50.8 (37.4–59.5)	2 (22.2)	1	7	1	− **0.260 (**− **0.381; **− **0.138)**	94.1	< 0.001
HDL, mmol/L	10	6 (4–9)	958	23 (19–77)	48.7 (37.4–55.0)	2 (20)	1	8	1	− **0.054 (**− **0.101; **− **0.006)**	97.1	< 0.001
Triglycerides, mmol/L	13	6 (4–9)	1,019	21 (17–66.5)	49.9 (41.0–59.2)	2 (15.4)	1	12	0	− 0.047 (− 0.141; 0.046)	89.1	< 0.001
Apo A, g/L	2	–	178	–	–	0 (0)	0	2	0	0.092 (0.042; 0.142)	0.0	0.780
Apo B, g/L	2	–	178	–	–	0 (0)	0	2	0	0.066 (− 0.257; 0.390)	97.1	< 0.001
Glucose homeostasis												
Glucose, mmol/L	9	8 (6–11.5)	717	21 (17.5–77)	54.4 (40.0–59.5)	1 (11.1)	0	9	0	0.021 (− 0.155; 0.198)	92.2	< 0.001
HbA1c, %	3	–	343	–	–	0 (0)	0	3	0	− **0.423 (**− **0.483; -0.364)**	0.0	0.760
Insulin, pmol/L	4	–	208	–	–	1 (25)	0	4	0	11.325 (− 4.220; 26.870)	68.7	0.010
Blood pressure												
Systolic blood pressure, mmHg	5	6 (5–8)	654	30 (20.7–44)	–	1 (20)	1	4	0	0.170 (− 2.168; 2.508)	88.3	< 0.001
Diastolic blood pressure, mmHg	5	6 (5–8)	654	30 (20.7–44)	–	1 (20)	1	4	0	− **1.154 (**− **2.030; -0.278)**	55.9	0.060

Significant weighted mean differences are bolded; *BMI* body mass index; *HbA1c* Glycated haemoglobin; *IQR* interquartile range; *WMD* Weighted mean difference *I*^2^ variation across studies that is due to heterogeneity rather than chance. *H* High risk of bias; *L* Low risk of bias; *SC* some concerns for bias

#### Subgroup analysis, leave-one-out analysis and publication bias

In subgroup analyses and meta-regression, only geographic location and sex were identified as potential sources of heterogeneity for TC and LDL-C analysis, respectively, (Supplemental Table 2). The leave-one-out analyses showed that findings on TC, HDL-C, LDL-C, TGs and glucose were not driven by any single study (Supplemental Tables 7–11). Regressing continuous variables, such as age, duration of study and number of study participants, on WMD showed some evidence of linear trends between percentage of male individuals and WMD of HDL-C and LDL-C. With increasing proportions of male participants, WMD of LDL-C (*p* = 0.03) and TC (*p* = 0.51) tended to decrease, but WMD of HDL-C increased (*p* = 0.007), Supplemental Figs. 5–8. No evidence was found for publication bias of RCTs included in meta-analysis comprising five or more estimates (Supplemental Figs. 17–22).

### Meta-analysis of RCTs comparing oat supplementation intervention versus heterogeneous control arms

A separate meta-analysis was performed including only 35 RCTs comparing CVD risk marker changes in an OSI vs. heterogeneous controls (e.g., rice, eggs, fibre, wheat, etc.). Results on blood lipid parameters remained similar to the other two meta-analyses, showing lowered TC and LDL-C in an OSI group compared to the control arms (Table 4). In addition, TGs [WMD: − 0.06 mmol/L, 95% CI (− 0.10; − 0.02)] and apolipoprotein B [WMD: − 0.03 g/L, 95% CI (− 0.05; − 0.01)] were significantly lower in the OSI arm compared to the control arm (Table 4). We found no differences for the rest of the investigated risk markers (Table 4).

**Table 4:** Meta-analysis of randomized clinical comparing oat supplementation intervention versus heterogeneous control arms

Outcome	Included studies		Participants			Study quality				Meta-analysis results		
	No. of unique studies	Follow-up duration, median (IQR), weeks	Total, no	Median sample size per intervention arm (IQR)	Age, median (IQR), years	No. of studies including healthy individuals, no. (%)	H	SC	L	WMD (95% CI)	*I*^2^ (%)	*P* value for heterogeneity
Body morphology												
BMI, kg/m^2^	13	6 (4.5–8)	862	25 (19.7–39.7)	53.2 (49.4–56.2)	2 (15)	1	12	0	**− 0.014 (− 0.220; 0.192)**	76.8	< 0.001
Body weight, kg	8	4 (3–9)	344	12 (10.7–28)	58.6 (43–62)	1 (12.5)	0	8	0	0.118 (**− **0.513; 0.749)	0.0	0.920
Waist circumference, cm	7	6 (4–13)	618	30.5 (24–66)	51.1 (33.9–55.8)	1 (14.3)	0	7	0	0.124 (**− **1.412; 1.660)	95.8	< 0.001
Body fat, %	3	–	149	–	–	0 (0)	0	3	0	1.020 (**− **1.957; 3.998)	92.3	< 0.001
Blood lipids												
Total cholesterol, mmol/L	28	5 (3.7–8)	1,922	24 (18–44.5)	53 (47.9–56.9)	6 (21.4)	3	25	0	**− 0.267 (− 0.385; -0.149)**	99.0	< 0.001
LDL, mmol/L	26	5 (4–8)	1,823	25 (16.5–47.2)	53 (48.1–56.6)	6 (46.1)	3	23	0	**− 0.163 (− 0.216; − 0.109)**	95.4	< 0.001
HDL, mmol/L	27	5 (4–8)	1,652	25 (18–48)	53 (48.1–56.6)	5 (18.5)	3	24	0	0.002 (**− **0.022; 0.025)	95.7	< 0.001
Triglycerides, mmol/L	26	5 (4–8)	1,802	24 (16.5–47.2)	53 (48.4–57.3)	4 (15.4)	3	23	0	**− 0.084 (− 0.153; − 0.015)**	98.7	< 0.001
Apo A, g/L	6	6 (5–8)	634	36.5 (19.7–74.5)	51.8 (48–54.5)	1 (16.7)	1	5	0	**− **0.005 (**− **0.029; 0.018)	89.0	< 0.001
Apo B, g/L	6	7 (5–11.7)	777	50 (20–7)	51.8 (49.0–52.8)	1 (16.7)	1	5	0	**− 0.031 (− 0.052; -0.010)**	98.8	< 0.001
Glucose homeostasis												
Glucose, mmol/L	15	6 (4–10)	1,142	21.5 (18.2–79)	59 (37.4–59.5)	1(6.7)	0	15	0	**− **0.009 (**− **0.064; 0.045)	93.6	< 0.001
HbA1c, %	2	–	113	–	–	0(0)	0	2	0	**− **0.076 (**− **0.321; 0.169)	87.7	0.004
Insulin, pmol/L	10	12 (5–12)	644	20 (12–36)	49.4 (41.7–55)	2 (20)	1	9	0	**− **0.641 (**− **4.218; 2.935)	82.7	< 0.001
Blood pressure												
Systolic blood pressure, mmHg	7	6 (4–8)	618	48 (19–66)	51.1 (33.9–55.8)	1 (12.5)	0	7	0	0.547 (**− **0.564; 1.657)	85.3	< 0.001
Diastolic blood pressure, mmHg	7	6 (4–8)	618	48 (19–66)	51.1 (33.9–55.8)	1 (12.5)	0	7	0	0.357 (**− **1.210; 1.925)	96.8	< 0.001

Significant weighted mean differences are bolded; *BMI* body mass index; *HbA1c* Glycated haemoglobin; *IQR* interquartile range; *WMD* Weighted mean difference *I*^2^ variation across studies that is due to heterogeneity rather than chance; *H* High risk of bias; *L* Low risk of bias; *SC* some concerns for bias

### Qualitative data synthesis

The scarcity of studies and the diversity of control arms across trials did not permit a meta-analysis of inflammation and oxidative stress markers. A summary of these results is available in Table 1.

In one study [20], daily supplementation of the diet with oat porridge containing 3 g of beta-glucan, among hypercholesterolemic adults, for 4 weeks resulted in decreased inflammatory marker levels, including high sensitivity C–reactive protein (hsCRP), interleukin 8 (IL-8), IL-6, and tumour necrosis factor alpha (TNF-α). The OSI also increased antioxidant capacities, by increasing the oxygen radical absorbance capacity and ferric reducing ability of plasma. Consumption of rice porridge did not lead to significant changes in these measures [20]. Oat interventions differ by botanical origin, molar mass, food matrix or degree of purification, and thus may have different effects on inflammatory markers [21]. In a trial including 75 hypercholesterolemic subjects receiving either 6 g/d concentrated OβGREs or 6 g/d dextrose (control) over a 6-week period, hsCRP did not significantly change in response to OβGREs [22]. Similarly, in an RCT comparing a mixture of wheat and oats with wheat only, none of the treatments significantly affected hsCRP or IL-6 [23]. In 43 otherwise healthy men and women with increased cholesterol levels, who consumed a daily ready-meal soup low in energy and fat and high in fibre, but with OβGREs vs. the same soup without OβGREs, there were no statistically significant changes observed in hsCRP between groups [24]. A single study on the antioxidant effects of avenanthramides was found: healthy people were randomized to the OSI group with oats-derived avenanthramides capsules (containing 3.12 mg avenanthramides) or placebo (corn oil capsules) or control group (no avenanthramides) for 1 month. Reported post-treatment serum levels of superoxide dismutase and reduced glutathione were found to significantly increase by 8.4% and 17.9%, respectively (*p* < 0.05) [25]. While malondialdehyde level significantly decreased by 28.1%, TC, TG and LDL-C levels were lowered by 11.1%, 28.1%, and 15.1% compared to no oats and control groups, respectively.

## Discussion

**Fig. 2:**
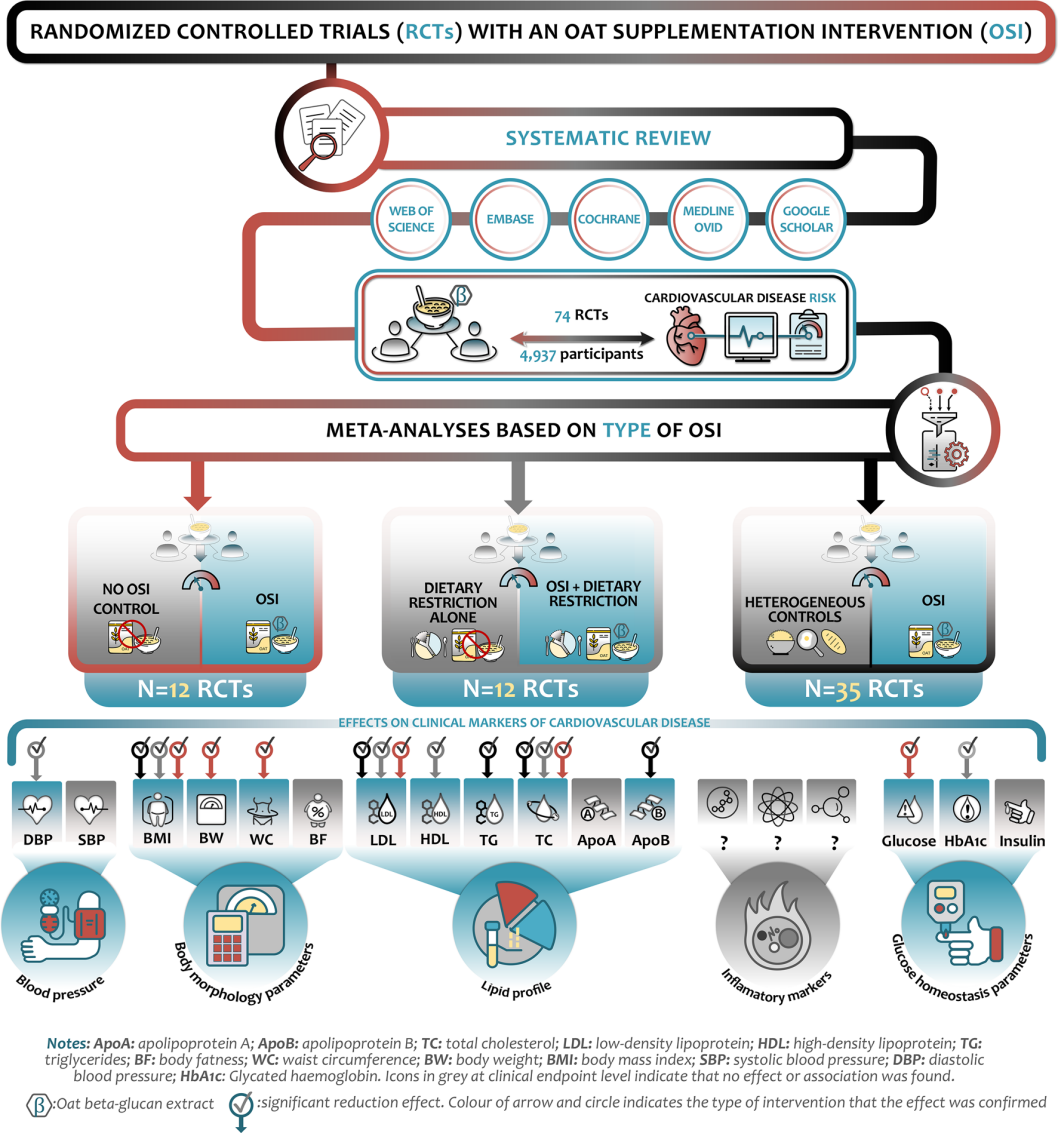
Graphical summary of main findings

In this systematic review and meta-analysis, dietary OSIs were associated with some improvements in a subset of CVD risk markers independently of the dietary background or control arm (Fig. 2). In particular, OSIs showed consistent decreases for BMI, total and LDL-C levels, regardless of the background diet or comparison group. OSIs lowered levels of HbA1c, diastolic BP and HDL-C only when compared to no OSIs. Furthermore, compared to heterogeneous control arms, potential benefits of oat dietary supplementation on apolipoprotein B and TG levels were observed, in addition to improved TC and LDL-C levels. A network meta-analysis has also suggested that OSIs can help regulate TC and LDL-C, indicating that increasing oat sources of whole grain may be recommended for lipid control [26].

Findings of meta-analyses have shown that intake of oat products can lower blood lipids, mainly serum LDL-C concentrations, but with a relatively modest reductions, which were variable within the range of real-world intakes. The role of oat products on lipid profile has been extensively studied in previous meta-analyses of RCTs, involving normal or mildly hypercholesterolaemic adults [6, 11, 27, 28]. Our meta-analysis included a larger number of studies, stratified the effects of an OSI by whether it was combined with another dietary restriction and demonstrated the beneficial effects of an OSI despite background diet or control arm.

Oats can exert health benefits via bioactive phytochemicals with potent antioxidant and anti-inflammatory effects, such as phytosterols, tocols, flavonoids, avenanthramides and soluble fibres such as beta-glucans [29, 30]. The cholesterol-lowering effects of soluble fibres can be partially explained by the modulating effect on absorption and re-absorption of cholesterol and bile acids due to their binding to fibre [31], or by the increased viscosity [32], which may modify the process of mixing, diffusion and/or emulsification in the gastrointestinal tract [33]. Soluble viscous fibres can influence dietary lipid metabolism in the mildly acidic medium found in the stomach [34]. Further, OβGREs have been shown to lower insulin release, which in turn can lower serum cholesterol levels [35]. Propionate produced by fermentation in the colon may inhibit cholesterol synthesis in the liver [35, 36]. This systemic interplay of oat bioactive phytochemicals and soluble fibres such as beta-glucans could have the potential to influence cardiometabolic health directly and indirectly, which warrants further investigation [4].

Apart from the lowering effect of an OSI on TC and LDL-C, a significant decrease in HDL-C was observed in the meta-analysis of RCTs comparing OSI + DR vs. DR alone. A recent RCT [37] has reported a similar HDL-C-lowering effect among patients with metabolic syndrome, in line with an RCT in 2010 [38]. This decrease in HDL-C may be linked to the background diet in the OSI group, which may have been unfavourable or influenced by confounding factors. Clinical and epidemiologic studies have established the presence of an inverse relationship between HDL-C levels and CVD risk, assuming that increased HDL-C levels are linked to protective effects on CVD [39, 40]. However, there is no sufficient evidence to show cardiovascular benefit of an OSI in patients on cholesterol-lowering therapy (e.g., statins), suggesting that HDL-C increases may not be sufficient to influence CVD risk, when LDL-C is kept in relatively low levels [41–43]. In addition, most research on HDL-C and Mendelian randomization studies have failed to find a direct effect of HDL-C on CVDs [41, 44]. However, it is reasonable to assume that we cannot ascertain the cause of this decrease in HDL-C and the role it may have on assessing the overall impact of oat intake on CVD risk. Future studies should explore how oat intake may affect different types of HDL-C particles, such as small-sized HDL-C, as well as their implications on cardiometabolic health [45].

A growing body of epidemiological studies [46–51] has consistently shown an inverse relationship between dietary fibre intake (such as those found in oats) and body weight. This report found a significant change in BMI, body weight and WC in the main meta-analysis. We observed similar effects and direction for BMI in the pooled analyses of OSIs + DR vs. DR alone. These findings suggest that the extent of health effects of an OSI on body morphology may be highly dependent on the background diet. When considering the effects of OSIs on BMI, body weight and/or WC, it is important to consider EFSA’s scientific requirements for health claims related to such parameters [52]. In particular, it should be taken into consideration that the duration of an intervention required to evaluate body weight should be at least 12 weeks and imaging data by established techniques (e.g., dual energy X-ray absorptiometry, magnetic resonance imaging or computed tomography) are generally appropriate to assess changes in body composition in human intervention studies. In our systematic review there were 20 RCTs with a duration of 12 weeks or more. In addition, not all interventions in RCTs were isocaloric, thus limiting our understanding of the impact of OSIs on obesity. Future clinical trials are needed to help address this question.

Effects of an OSI on BP were only observed for diastolic BP, in the case of OSI + DR vs. DR alone. This change (i.e., WMD: − 1.15 mm Hg, 95% CI (− 2.03; − 0.28)) was inconsistent in other types of interventions and not found in case of systolic BP. A similar inconsistency was observed for glucose homeostasis markers, where significant differences were observed for HbA1c only in RCTs comparing OSI + DR versus DR alone and for glucose for RCTs comparing OSIs vs. no OSIs. No significant differences were observed in any other intervention or in interventions comparing an OSI with heterogeneous controls, regarding any glucose homeostasis marker. A meta-analysis of RCTs evaluating the effects of oat products on glycaemic control among diabetic patients indicated that the effects of oats and oat beta-glucans on glycaemic control and insulin sensitivity are inconclusive [5]. In line with our work, a systematic review on oat intake and its association with CVD risk markers did not find convincing evidence of oat influence on insulin sensitivity and emphasized the importance of exploring additional CVD markers [4]. However, it has been proposed that the glycaemic benefits of oats are directly dependent on the structural integrity of the oat kernel, β-glucan’s dose, molecular weight and comparison [13, 53–55]. Even though our findings were based on a limited number of studies focused on OSIs and glucose homeostasis, they still suggest some benefits for the later and thus warrant the need for further more rigorous research.

### Strengths, limitations and recommendations for future research

To the best of our knowledge, this is the first study to provide a comprehensive analysis on the role of OSI on several CVD risk markers, accounting for background diet and control arms. To identify as many relevant studies as possible and reduce the risk of publication bias, a highly sensitive search strategy was used and additional resources were searched including the reference lists of included trials and relevant systematic reviews. Conventional funnel plots and Egger test estimates showed only a minimal publication bias; still, these methods are limited by their qualitative nature and we cannot exclude the possibility of measured or unmeasured publication bias. Location of study and percentage of male participants contributed to the heterogeneity of findings, and the OSI’s dose and duration were highly variable. Thus, future studies exploring the role of sex, ethnicity and cultural factors in the association of OSIs and risk of CVD are warranted. Our findings need to be interpreted cautiously, with considerations of the specific comparison food/diet. Also, only 36 out of 74 RCTs (48.6%) took isocaloric diet between arms into account, and whether these differences affect the results should be explored by future studies.

## Conclusion

Supplementation of diet with oat cereals improves CVD risk markers among healthy adults and those with mild metabolic disturbances, particularly by influencing serum total and LDL cholesterol, BMI and WC. The beneficial effects on TC and LDL-C were independent of the dietary background. The role of OSIs on BP, glucose homeostasis or other markers, could not be established.

## Supplementary Information

Below is the link to the electronic supplementary material.

Supplementary file1 (DOCX 323 KB)
