# Supplementary material for: Effect of oat supplementation interventions on cardiovascular disease risk markers: a systematic review and meta-analysis of randomized controlled trials
Source: Eur J Nutr. 2022 Jan 3;61(4):1749–78. doi: 10.1007/s00394-021-02763-1 (PMC9106631; doi:10.1007/s00394-021-02763-1)
Supplement: Supplementary file 1 — Supplementary file1 (DOCX 323 KB) [file 394_2021_2763_MOESM1_ESM.docx]

**Table of Contents**

[Search Strategy 2](#_Toc82172237)

[Supplementary table 1. Overview databases and results (Date last searched: May 15, 2020) 2](#_Toc82172238)

[Search string for Embase 3](#_Toc82172239)

[Search string for Medline Ovid 3](#_Toc82172240)

[Search string for Cochrane 4](#_Toc82172241)

[Search string for Web of Science 4](#_Toc82172242)

[Search string for Google scholar (first 200 out of 241’000) 4](#_Toc82172243)

[Subgroup analyses 5](#_Toc82172244)

[Supplemental table 2. Subgroup analyses 5](#_Toc82172245)

[Leave one out sensitivity analysis 6](#_Toc82172246)

[Supplemental table 3. Leave one out sensitivity analysis for total cholesterol, in RCTs comparing OSIs vs. no OSIs controls 6](#_Toc82172247)

[Supplemental table 4. Leave one out sensitivity analysis for HDL, in RCTs comparing OSIs vs. no OSIs controls 6](#_Toc82172248)

[Supplemental table 5. Leave one out sensitivity analysis for LDL, in RCTs comparing OSIs vs. no OSIs controls 6](#_Toc82172249)

[Supplemental table 6. Leave one out sensitivity analysis for triglycerides comparing OSIs vs. no OSIs controls 7](#_Toc82172250)

[Supplemental table 7. Leave one out sensitivity analysis for total cholesterol, in RCTs comparing OSIS+DR vs. DR alone 7](#_Toc82172251)

[Supplemental table 8. Leave one out sensitivity analysis for HDL, in RCTs comparing OSIS+DR vs. DR alone 7](#_Toc82172252)

[Supplemental table 9. Leave one out sensitivity analysis for LDL, in RCTs comparing OSIS+DR vs. DR alone 7](#_Toc82172253)

[Supplemental table 10. Leave one out sensitivity analysis for triglycerides, in RCTs comparing OSIS+DR vs. DR alone 8](#_Toc82172254)

[Supplemental table 11. Leave one out sensitivity analysis for glucose, in RCTs comparing OSIS+DR vs. DR alone 8](#_Toc82172255)

[Linear meta-regression analysis 9](#_Toc82172256)

[Supplemental figure 1 (a-d). Linear meta-regression analysis for total cholesterol for RCTs comparing OSIs vs. no OSIs controls 9](#_Toc82172257)

[Supplemental figure 2 (a-d). Linear meta-regression analysis for HDL cholesterol for RCTs comparing OSI vs no OSI controls 9](#_Toc82172258)

[Supplemental figure 3 (a-d). Linear meta-regression analysis for LDL cholesterol for RCTs comparing OSI vs no OSI controls 10](#_Toc82172259)

[Supplemental figure 4 (a-d). Linear meta-regression analysis for triglycerides for RCTs comparing OSI vs no OSI controls 10](#_Toc82172260)

[Supplemental figure 5 (a-d). Linear meta-regression analysis for total cholesterol for RCTs comparing OSIS+DR vs. DR alone 11](#_Toc82172261)

[Supplemental figure 6 (a-d). Linear meta-regression analysis for HDL cholesterol for RCTs comparing OSIS+DR vs. DR alone 11](#_Toc82172262)

[Supplemental figure 7 (a-d). Linear meta-regression analysis for LDL cholesterol for RCTs comparing OSIS+DR vs. DR alone 12](#_Toc82172263)

[Supplemental figure 8 (a-d). Linear meta-regression analysis for triglycerides for RCTs comparing OSIS+DR vs. DR alone 12](#_Toc82172264)

[Publication bias analysis 13](#_Toc82172265)

[Supplemental figure 9. Publication bias for RCTs comparing OSIs vs. no OSIs controls for total cholesterol 13](#_Toc82172266)

[Supplemental figure 10. Publication bias for RCTs comparing OSIs vs. no OSIs controls for HDL cholesterol 13](#_Toc82172267)

[Supplemental figure 11. Publication bias for RCTs comparing OSIs vs. no OSIs controls for LDL cholesterol 14](#_Toc82172268)

[Supplemental figure 12. Publication bias for RCTs comparing OSIs vs. no OSIs controls for triglycerides 14](#_Toc82172269)

[Supplemental figure 13. Publication bias for RCTs comparing OSIs vs. no OSIs controls for body mass index 15](#_Toc82172270)

[Supplemental figure 14. Publication bias for RCTs comparing OSIs vs. no OSIs controls for body weight 15](#_Toc82172271)

[Supplemental figure 15. Publication bias for RCTs comparing OSIs vs. no OSIs controls for systolic blood pressure 15](#_Toc82172272)

[Supplemental figure 16. Publication bias for RCTs comparing OSIs vs. no OSIs controls for diastolic blood pressure 16](#_Toc82172273)

[Supplemental figure 17. Publication bias for RCTs comparing OSIs+DR vs. DR alone, for body mass index 16](#_Toc82172274)

[Supplemental figure 18. Publication bias for RCTs comparing OSIs+DR vs. DR alone, for total body weight 16](#_Toc82172275)

[Supplemental figure 19. Publication bias for RCTs comparing OSIs+DR vs. DR alone, for total waist circumference 17](#_Toc82172276)

[Supplemental figure 20. Publication bias for RCTs comparing OSIs+DR vs. DR alone, for total glucose 17](#_Toc82172277)

[Supplemental figure 21. Publication bias for RCTs comparing OSIs+DR vs. DR alone, for total systolic blood pressure 18](#_Toc82172278)

[Supplemental figure 22. Publication bias for RCTs comparing OSIs+DR vs. DR alone, for diastolic blood pressure 18](#_Toc82172279)

# Search Strategy

**Search strategies for the CV risk factors are mainly based on the strategies developed by Wichor M. Bramer, supplementary material: Asllanaj E, Zhang X, Ochoa Rosales C, Nano J, Bramer WM, Portilla-Fernandez E,Braun KVE, Gonzalez-Jaramillo V, Ahrens W, Ikram A, Ghanbari M, Voortman T, Franco OH, Muka T, Glisic M. Sexually dimorphic DNA-methylation in cardiometabolic health: A systematic review. Maturitas. 2020 May;135:6-26. doi:10.1016/j.maturitas.2020.02.005. Epub 2020 Feb 13. Review. PubMed PMID: 32252966.*

## Supplementary table 1a. Overview databases and results (Date last searched: May 15, 2020)

|  | **Before deduplication** | **After deduplication** |
| --- | --- | --- |
| Embase | 1506 | 1489 |
| Medline Ovid | 1314 | 334 |
| Cochrane | 1463 | 667 |
| Web of Science | 1292 | 626 |
| Google scholar | 200 | 123 |
| Total | **5775** | **3239** |

*2536 duplicated records have been removed*

| **Oat(s)** |
| --- |
| ('oat'/de OR 'oat bran'/de OR 'beta glucan'/de OR 'whole grain'/de OR ('avena sativa' OR oat OR oats OR oatmeal* OR oatcake* OR porridge* OR muesli OR granola OR b-glucan* OR β-glucan* OR beta-glucan* OR beta-dextroglucan* OR 'whole grain*' OR wholegrain*):ab,ti,kw) |
| **Glucose** |
| ('insulin response'/exp OR 'glucose blood level'/exp OR 'insulin blood level'/exp OR hyperinsulinism/exp OR 'impaired glucose tolerance'/de OR 'impaired fasting glucose'/de OR 'hyperglycemia'/de OR 'fasting glucose'/de OR 'glycosylated hemoglobin'/exp OR 'glycemic control'/de OR 'homa ir'/exp OR (((glucose OR sugar OR insulin*) NEAR/3 (level* OR blood OR serum OR plasma* OR concentration* OR tolerance OR intolerance OR sensitiv* OR insensitiv* OR resistan* OR response OR dependen* OR homeosta*)) OR hypoglycemi* OR hypoglycaemi* OR hyperglycemi* OR hyperglycaemi* OR antihyperglycemi* OR prediabet* OR pre-diabet* OR fasting NEAR/2 glucose OR 'glycated hemoglobin' OR 'glycated haemoglobin' OR glycosylated haemoglobin OR glycosylated hemoglobin OR HbA1c OR 'Hb A1c' OR 'hemoglobin A1c' OR 'haemoglobin A1c' OR HOMA-IR OR glycaem* OR glycem* OR glucosaemia OR glucosemia OR hyperinsulin* OR hypoinsulin* OR insulinem* OR insulinaem* OR prediabet* OR pre-diabet*):ab,ti,kw) |
| **Obesity** |
| (obesity/exp OR 'body mass'/de OR 'body fat'/de OR 'waist circumference'/de OR 'waist hip ratio'/de OR (obes* OR 'over weight' OR overweight OR adipos* OR 'body mass' OR BMI OR ((abdom* OR body) NEAR/3 (fat OR weight OR composition OR size)) OR ('weight gain*') OR ('weight loss*') OR (fat NEAR/3 (mass OR percentage* OR distribution)) OR (waist NEAR/3 (hip OR circumference*))):ab,ti,kw) |
| **Blood pressure** |
| ('abnormal blood pressure'/exp OR 'blood pressure'/exp OR (hypertensi* OR hypotensi* OR 'blood pressure*'):ab,ti,kw) |
| **Blood Lipids** |
| ('lipid blood level'/exp OR 'low density lipoprotein cholesterol'/de OR 'high density lipoprotein cholesterol'/exp OR 'triacylglycerol'/de OR (((lipid* OR cholester* OR triacylglycerol* OR triglyceride* OR HDL* OR LDL* OR VLDL* OR VHDL*) NEAR/6 (level* OR blood OR serum OR plasma* OR concentration*)) OR hypercholesterol*):ab,ti,kw) |
| **Atherosclerosis** |
| (atherosclerosis/de OR 'atherosclerotic plaque'/de OR 'carotid atherosclerosis'/exp OR 'coronary artery atherosclerosis'/de OR 'arterial wall thickness'/de OR 'coronary artery calcification'/de OR (atherosclero* OR ((intima-media OR intimamedia ) NEAR/3 thickness*) OR ((arterial) NEAR/1 (wall OR thickness)) OR (coronar* NEAR/3 calcif*)):ab,ti,kw) |
| **Inflammation** |
| ((inflammation/de AND (marker/de OR 'C reactive protein'/exp OR cytokine/de OR fibrinolysis/exp OR 'tumor necrosis factor alpha'/exp)) OR 'chronic inflammation'/exp OR 'oxidative stress'/de OR ((inflammat* NEAR/3 (chronic* OR marker* OR biomarker* OR interleukin* OR crp OR 'c reactive' OR cytokine* OR fibrinolys* OR fibrinogenlys* OR 'tumor necrosis factor' OR tnf )) OR (oxidative NEAR/3 stress*)):ab,ti,kw) |
| **General cardiovascular diseases-risk factors, cardio-metabolic risk factors, arterial stiffness, metabolic syndrome** |
| ('cardiovascular risk'/exp OR 'cardiometabolic risk'/de OR 'Metabolic Syndrome X'/de OR 'arterial stiffness'/de OR (((cardiovascular OR cardiometabol* OR cardio-metabol* OR metabolic) NEAR/3 (risk* OR syndrome*)) OR CV-risk OR ((vascular OR arterial) NEAR/1 (stiffness))):ab,ti,kw) |

**Limits**

Exclusion: NOT ([animals]/lim NOT [humans]/lim) NOT ([Conference Abstract]/lim OR [Letter]/lim OR [Note]/lim OR [Editorial]/lim)

Filters for RCTs (based on Cochrane, with adaptions by Wichor Bramer)

## Search string for EMBASE

('oat'/de OR 'oat bran'/de OR 'beta glucan'/de OR 'whole grain'/de OR ('avena sativa' OR oat OR oats OR oatmeal* OR oatcake* OR porridge* OR muesli OR granola OR b-glucan* OR β-glucan* OR beta-glucan* OR beta-dextroglucan* OR 'whole grain*' OR wholegrain*):ab,ti,kw) AND (('insulin response'/exp OR 'glucose blood level'/exp OR 'insulin blood level'/exp OR hyperinsulinism/exp OR 'impaired glucose tolerance'/de OR 'impaired fasting glucose'/de OR 'hyperglycemia'/de OR 'fasting glucose'/de OR 'glycosylated hemoglobin'/exp OR 'glycemic control'/de OR 'homa ir'/exp OR (((glucose OR sugar OR insulin*) NEAR/3 (level* OR blood OR serum OR plasma* OR concentration* OR tolerance OR intolerance OR sensitiv* OR insensitiv* OR resistan* OR response OR dependen* OR homeosta*)) OR hypoglycemi* OR hypoglycaemi* OR hyperglycemi* OR hyperglycaemi* OR antihyperglycemi* OR prediabet* OR pre-diabet* OR fasting NEAR/2 glucose OR 'glycated hemoglobin' OR 'glycated haemoglobin' OR glycosylated haemoglobin OR glycosylated hemoglobin OR HbA1c OR 'Hb A1c' OR 'hemoglobin A1c' OR 'haemoglobin A1c' OR HOMA-IR OR glycaem* OR glycem* OR glucosaemia OR glucosemia OR hyperinsulin* OR hypoinsulin* OR insulinem* OR insulinaem* OR prediabet* OR pre-diabet*):ab,ti,kw) OR (obesity/exp OR 'body mass'/de OR 'body fat'/de OR 'waist circumference'/de OR 'waist hip ratio'/de OR (obes* OR 'over weight' OR overweight OR adipos* OR 'body mass' OR BMI OR ((abdom* OR body) NEAR/3 (fat OR weight OR composition OR size)) OR ('weight gain*') OR ('weight loss*') OR (fat NEAR/3 (mass OR percentage* OR distribution)) OR (waist NEAR/3 (hip OR circumference*))):ab,ti,kw) OR ('abnormal blood pressure'/exp OR 'blood pressure'/exp OR (hypertensi* OR hypotensi* OR 'blood pressure*'):ab,ti,kw) OR ('lipid blood level'/exp OR 'low density lipoprotein cholesterol'/de OR 'high density lipoprotein cholesterol'/exp OR 'triacylglycerol'/de OR (((lipid* OR cholester* OR triacylglycerol* OR triglyceride* OR HDL* OR LDL* OR VLDL* OR VHDL*) NEAR/6 (level* OR blood OR serum OR plasma* OR concentration*)) OR hypercholesterol*):ab,ti,kw) OR (atherosclerosis/de OR 'atherosclerotic plaque'/de OR 'carotid atherosclerosis'/exp OR 'coronary artery atherosclerosis'/de OR 'arterial wall thickness'/de OR 'coronary artery calcification'/de OR (atherosclero* OR ((intima-media OR intimamedia ) NEAR/3 thickness*) OR ((arterial) NEAR/1 (wall OR thickness)) OR (coronar* NEAR/3 calcif*)):ab,ti,kw) OR ((inflammation/de AND (marker/de OR 'C reactive protein'/exp OR cytokine/de OR fibrinolysis/exp OR 'tumor necrosis factor alpha'/exp)) OR 'chronic inflammation'/exp OR 'oxidative stress'/de OR ((inflammat* NEAR/3 (chronic* OR marker* OR biomarker* OR interleukin* OR crp OR 'c reactive' OR cytokine* OR fibrinolys* OR fibrinogenlys* OR 'tumor necrosis factor' OR tnf )) OR (oxidative NEAR/3 stress*)):ab,ti,kw) OR ('cardiovascular risk'/exp OR 'cardiometabolic risk'/de OR 'Metabolic Syndrome X'/de OR 'arterial stiffness'/de OR (((cardiovascular OR cardiometabol* OR cardio-metabol* OR metabolic) NEAR/3 (risk* OR syndrome*)) OR CV-risk OR ((vascular OR arterial) NEAR/1 (stiffness))):ab,ti,kw)) NOT ([animals]/lim NOT [humans]/lim) NOT ([Conference Abstract]/lim OR [Letter]/lim OR [Note]/lim OR [Editorial]/lim) AND ('clinical trial'/exp OR 'randomization'/de OR 'Crossover procedure'/de OR 'Double-blind procedure'/de OR 'Single-blind procedure'/de OR (random* OR factorial* OR crossover* OR (cross NEXT/1 over*) OR placebo* OR ((doubl* OR singl*) NEXT/1 blind*) OR assign* OR allocat* OR volunteer* OR trial OR groups OR rct):ab,ti)

## Search string for MEDline (Ovid)

(Avena/ OR exp beta-Glucans/ OR Whole Grains/ OR (avena sativa OR oat OR oats OR oatmeal* OR oatcake* OR porridge* OR muesli OR granola OR b-glucan* OR beta-glucan* OR beta-dextroglucan* OR whole grain* OR wholegrain*).ab,ti,kw.) AND ((Insulin Resistance/ OR glucose/bl OR insulin/bl OR hyperinsulinism/ OR exp Hyperglycemia/ OR Glycated Hemoglobin A/ OR (((glucose OR sugar OR insulin*) ADJ3 (level* OR blood OR serum OR plasma* OR concentration* OR tolerance OR intolerance OR sensitiv* OR insensitiv* OR resistan* OR response OR dependen* OR homeosta*)) OR hypoglycemi* OR hypoglycaemi* OR hyperglycemi* OR hyperglycaemi* OR antihyperglycemi* OR prediabet* OR pre-diabet* OR fasting ADJ2 glucose OR glycated hemoglobin OR glycated haemoglobin OR glycosylated haemoglobin OR glycosylated hemoglobin OR HbA1c OR Hb A1c OR hemoglobin A1c OR haemoglobin A1c OR HOMA-IR OR glycaem* OR glycem* OR glucosaemia OR glucosemia OR hyperinsulin* OR hypoinsulin* OR insulinem* OR insulinaem* OR prediabet* OR pre-diabet*).ab,ti,kw.) OR (exp obesity/ OR "Body Mass Index"/ OR exp abdominal fat/ OR exp Body Weights and Measures/ OR exp Body Composition/ OR (obes* OR over weight OR overweight OR adipos* OR body mass OR BMI OR ((abdom* OR body) ADJ3 (fat OR weight OR composition OR size)) OR (weight gain*) OR (weight loss*) OR (fat ADJ3 (mass OR percentage* OR distribution)) OR (waist ADJ3 (hip OR circumference*))).ab,ti,kw.) OR (exp Hypertension/ OR exp Hypotension/ OR exp blood pressure/ OR (hypertensi* OR hypotensi* OR blood pressure*).ab,ti,kw.) OR (exp lipids/bl OR (((lipid* OR cholester* OR triacylglycerol* OR triglyceride* OR HDL* OR LDL* OR VLDL* OR VHDL*) ADJ6 (level* OR blood OR serum OR plasma* OR concentration*)) OR hypercholesterol*).ab,ti,kw.) OR (atherosclerosis/ OR Plaque, Atherosclerotic/ OR Carotid Artery Diseases/ OR "Coronary Artery Disease"/ OR "Carotid Intima-Media Thickness"/ OR (atherosclero* OR ((intima-media OR intimamedia) ADJ3 thickness*) OR (coronar* ADJ3 calcif*)).ab,ti,kw.) OR ((inflammation/ AND (exp Biological Markers/ OR C-Reactive Protein/ OR cytokine/ OR fibrinolysis/ OR Tumor Necrosis Factor-alpha/)) OR Oxidative Stress/ OR ((inflammat* ADJ3 (chronic* OR marker* OR biomarker* OR interleukin* OR crp OR "c reactive" OR cytokine* OR fibrinolys* OR fibrinogenlys* OR "tumor necrosis factor" OR tnf)) OR (oxidative ADJ3 stress*)).ab,ti,kw.) OR (Metabolic Syndrome/ OR Vascular Stiffness/ OR (((cardiovascular OR cardiometabol* OR cardio-metabol* OR metabolic) ADJ3 (risk* OR syndrome*)) OR CV-risk OR ((vascular OR arterial) ADJ1 (stiffness))).ab,ti,kw.)) NOT (exp animals/ NOT humans/) NOT (letter* OR news OR comment* OR editorial* OR congres* OR abstract* OR book* OR chapter* OR dissertation abstract*).pt. AND (Exp Controlled clinical trial/ OR "Double-Blind Method"/ OR "Single-Blind Method"/ OR "Random Allocation"/ OR (random* OR factorial* OR crossover* OR (cross ADJ over*) OR placebo* OR ((doubl* OR singl*) ADJ blind*) OR assign* OR allocat* OR volunteer* OR trial OR groups OR rct).ab,ti.)

## Search string for Cochrane

("avena sativa" OR oat OR oats OR oatmeal* OR oatcake* OR porridge* OR muesli OR granola OR b-glucan* OR β-glucan* OR beta-glucan* OR beta-dextroglucan* OR "whole grain*" OR (whole NEXT grain*) OR wholegrain*):ab,ti,kw AND ((((glucose OR sugar OR insulin*) NEAR/3 (level* OR blood OR serum OR plasma* OR concentration* OR tolerance OR intolerance OR sensitiv* OR insensitiv* OR resistan* OR response OR dependen* OR homeosta*)) OR hypoglycemi* OR hypoglycaemi* OR hyperglycemi* OR hyperglycaemi* OR antihyperglycemi* OR prediabet* OR pre-diabet* OR fasting NEAR/2 glucose OR "glycated hemoglobin" OR "glycated haemoglobin" OR "glycosylated haemoglobin" OR "glycosylated hemoglobin" OR HbA1c OR "Hb A1c" OR "hemoglobin A1c" OR "haemoglobin A1c" OR HOMA-IR OR glycaem* OR glycem* OR glucosaemia OR glucosemia OR hyperinsulin* OR hypoinsulin* OR insulinem* OR insulinaem*):ab,ti,kw OR (obes* OR "over weight" OR overweight OR adipos* OR "body mass" OR BMI OR ((abdom* OR body) NEAR/3 (fat OR weight OR composition OR size)) OR (weight NEXT gain*) OR (weight NEXT loss*) OR (fat NEAR/3 (mass OR percentage* OR distribution)) OR (waist NEAR/3 (hip OR circumference*))):ab,ti,kw OR (hypertensi* OR hypotensi* OR blood NEXT pressure*):ab,ti,kw OR (((lipid* OR cholester* OR triacylglycerol* OR triglyceride* OR HDL* OR LDL* OR VLDL* OR VHDL*) NEAR/6 (level* OR blood OR serum OR plasma* OR concentration*)) OR hypercholesterol*):ab,ti,kw OR (atherosclero* OR ((intima-media OR intimamedia ) NEAR/3 thickness*) OR ((arterial) NEAR/1 (wall OR thickness)) OR (coronar* NEAR/3 calcif*)):ab,ti,kw OR ((inflammat* NEAR/3 (chronic* OR marker* OR biomarker* OR interleukin* OR crp OR "c reactive" OR cytokine* OR fibrinolys* OR fibrinogenlys* OR "tumor necrosis factor" OR tnf )) OR (oxidative NEAR/3 stress*)):ab,ti,kw OR (((cardiovascular OR cardiometabol* OR cardio-metabol* OR metabolic) NEAR/3 (risk* OR syndrome*)) OR CV-risk OR ((vascular OR arterial) NEAR/1 (stiffness))):ab,ti,kw)

## Search string for Web of Science

TS=(((("avena sativa" OR oat OR oats OR oatmeal* OR oatcake* OR porridge* OR muesli OR granola OR b-glucan* OR β-glucan* OR beta-glucan* OR beta-dextroglucan* OR "whole grain*" OR wholegrain*) AND ((((glucose OR sugar OR insulin*) NEAR/3 (level* OR blood OR serum OR plasma* OR concentration* OR tolerance OR intolerance OR sensitiv* OR insensitiv* OR resistan* OR response OR dependen* OR homeosta*)) OR hypoglycemi* OR hypoglycaemi* OR hyperglycemi* OR hyperglycaemi* OR antihyperglycemi* OR prediabet* OR pre-diabet* OR fasting NEAR/2 glucose OR "glycated hemoglobin" OR "glycated haemoglobin" OR "glycosylated haemoglobin" OR "glycosylated hemoglobin" OR HbA1c OR "Hb A1c" OR "hemoglobin A1c" OR "haemoglobin A1c" OR HOMA-IR OR glycaem* OR glycem* OR glucosaemia OR glucosemia OR hyperinsulin* OR hypoinsulin* OR insulinem* OR insulinaem*) OR (obes* OR "over weight" OR overweight OR adipos* OR "body mass" OR BMI OR ((abdom* OR body) NEAR/3 (fat OR weight OR composition OR size)) OR ("weight gain*") OR ("weight loss*") OR (fat NEAR/3 (mass OR percentage* OR distribution)) OR (waist NEAR/3 (hip OR circumference*))) OR (hypertensi* OR hypotensi* OR "blood pressure*") OR (((lipid* OR cholester* OR triacylglycerol* OR triglyceride* OR HDL* OR LDL* OR VLDL* OR VHDL*) NEAR/6 (level* OR blood OR serum OR plasma* OR concentration*)) OR hypercholesterol*) OR (atherosclero* OR ((intima-media OR intimamedia ) NEAR/3 thickness*) OR ((arterial) NEAR/1 (wall OR thickness)) OR (coronar* NEAR/3 calcif*)) OR ((inflammat* NEAR/3 (chronic* OR marker* OR biomarker* OR interleukin* OR crp OR "c reactive" OR cytokine* OR fibrinolys* OR fibrinogenlys* OR "tumor necrosis factor" OR tnf )) OR (oxidative NEAR/3 stress*)) OR (((cardiovascular OR cardiometabol* OR cardio-metabol* OR metabolic) NEAR/3 (risk* OR syndrome*)) OR CV-risk OR ((vascular OR arterial) NEAR/1 (stiffness))))) AND (random* OR placebo* OR trial OR rct))) AND DT=(article)

NOT TS=((animal* OR rat OR rats OR mouse OR mice OR hens OR murine OR sheep OR pigs OR nonhuman* OR primate*) NOT (human* OR patient*))

## Search string for Google scholar (first 200 out of 241’000)

avena|oat|oats|oatmeal|granola|glucan|"whole grain"|wholegrain|"whole grains"|wholegrains cardiovascular|coronary|cardiometabolic|vascular|glucose|insulin|obesity|BMI|overweight|"blood pressure"|hypertension|lipid|cholesterol risk|risks trial|study

# Subgroup analyses

## Supplemental table 2. Subgroup analyses

| **Study characteristics** | | **OSI vs. no OSI controls** | | **OSIs+DR vs. DR alone** | |
| --- | --- | --- | --- | --- | --- |
|  |  | **WMD and 95% CI** | **P value (meta-regression)** | **WMD and 95% CI** | **P value (meta-regression)** |
| **Total cholesterol, mmol/L** | | | | | |
| **Age** | ≤Median | -0.461 (-0.778;0.143) | 0.45 | -0.610 (-0.879;0.340) | 0.07 |
|  | >Median | -0.269 (-0.487;0.052) |  | -0.289 (-0.395;0.183) |  |
| **Sex** | ≤Median | -0.373 (-0.731;0.015) | 0.27 | -0.436 (-0.614;0.257) | 0.76 |
|  | >Median | -0.594 (-0.804;0.384) |  | -0.501 (-0.878;0.125) |  |
| **Health status** | Healthy | n. a. | 0.66 | n. a. | 0.75 |
|  | Others | -0.427 (-0.630;0.224) |  | -0.421 (-0.553;0.290) |  |
| **Intervention duration** | ≤Median | -0.261 (-0.400;0.122) | 0.19 | -0.535 (-0.712;0.359) | 0.11 |
|  | >Median | -0.524 (-0.855;0.193) |  | -0.249 (-0.323;0.174) |  |
| **Location** | North America | -0.281 (-0.534;0.029) | 0.45 | -0.737 (-1.097;0.377) | **0.02** |
|  | Europe | -0.383 (-0.847 0.080) |  | -0.670 (-1.023;0.316) |  |
|  | South America | n. a. |  | -0.248 (-0.352;0.144) |  |
|  | Other | -0.540 (-0.831;0.250) |  | -0.275 (-0.397;0.153) |  |
| **Participants** | ≤Median | -0.416 (-0.653;0.180) | 0.96 | -0.434 (-0.790;0.078) | 0.92 |
|  | >Median | -0.411 (-0.707;0.115) |  | -0.378 (-0.499;0.256) |  |
| **LDL, mmol/L** | | | | | |
| **Age** | ≤Median | -0.245 (-0.323;0.167) | 0.52 | -0.107 (-0.273 0.058) | 0.07 |
|  | >Median | -0.328 (-0.559;0.098) |  | -0.427 (-0.603;0.252) |  |
| **Sex** | ≤Median | -0.147 (-0.238;0.056) | **0.02** | -0.233 (-0.550 0.085) | 0.63 |
|  | >Median | -0.413 (-0.559;0.266) |  | -0.320 (-0.479;0.161) |  |
| **Health status** | Healthy | n. a. | 0.62 | -0.237 (-0.569 0.096) | 0.91 |
|  | Others | -0.294 (-0.386;0.202) |  | -0.264 (-0.397;0.130) |  |
| **Intervention duration** | ≤Median | -0.237 (-0.350;0.124) | 0.34 | -0.355 (-0.509;0.200) | 0.19 |
|  | >Median | -0.344 (-0.499;0.189) |  | -0.123 (-0.402 0.156) |  |
| **Location** | North America | -0.214 (-0.328;0.100) | 0.13 | -0.546 (-0.787;0.304) | 0.32 |
|  | Europe | -0.250 (-0.395;0.105) |  | -0.277 (-0.751 0.198) |  |
|  | South America | n. a. |  | -0.257 (-0.761 0.247) |  |
|  | Other | -0.420 (-0.610;0.229) |  | -0.137 (-0.272;0.002) |  |
| **Participants** | ≤Median | -0.332 (-0.486;0.177) | 0.4 | -0.292 (-0.523;0.060) | 0.72 |
|  | >Median | -0.233 (-0.331;0.135) |  | -0.194 (-0.328;0.059) |  |
| **HDL, mmol/L** | | | | | |
| **Age** | ≤Median | -0.008 (-0.048 0.033) | 0.50 | -0.182 (-0.347;0.016) | 0.21 |
|  | >Median | -0.039 (-0.084 0.006) |  | 0.010 (-0.111 0.130) |  |
| **Sex** | ≤Median | -0.009 (-0.054 0.035) | 0.47 | -0.192 (-0.339;0.044) | 0.47 |
|  | >Median | -0.046 (-0.074;0.018) |  | -0.364 (-0.537;0.191) |  |
| **Health status** | Healthy | n. a. | 0.18 | 0.030 (-0.166 0.226) | 0.50 |
|  | Others | -0.009 (-0.035 0.018) |  | -0.069 (-0.121;0.017) |  |
| **Intervention duration** | ≤Median | -0.007 (-0.045 0.031) | 0.65 | -0.010 (-0.050 0.031) | 0.27 |
|  | >Median | -0.022 (-0.063 0.018) |  | -0.153 (-0.350 0.044) |  |
| **Location** | North America | 0.004 (-0.066 0.074) | 0.55 | 0.042 (-0.134 0.218) | 0.43 |
|  | Europe | -0.027 (-0.070 0.015) |  | -0.091 (-0.140;0.042) |  |
|  | South America | n. a. |  | 0.067 (-0.057 0.191) |  |
|  | Other | -0.000 (-0.027 0.027) |  | -0.179 (-0.264;0.093) |  |
| **Participants** | ≤Median | -0.012 (-0.070 0.047) | 0.87 | -0.117 (-0.345 0.112) | 0.49 |
|  | >Median | -0.016 (-0.047 0.014) |  | -0.019 (-0.055 0.018) |  |
| **Triglycerides, mmol/L** | | | | | |
| **Age** | ≤Median | -0.058 (-0.141 0.026) | 0.13 | -0.046 (-0.094 0.003) | 0.32 |
|  | >Median | 0.081 (-0.036 0.198) |  | 0.004 (-0.175 0.183) |  |
| **Sex** | ≤Median | -0.032 (-0.166 0.102) | 0.77 | -0.092 (-0.189 0.005) | 0.16 |
|  | >Median | 0.004 (-0.171 0.179) |  | 0.037 (-0.131 0.205) |  |
| **Health status** | Healthy | n. a. | n. a. | -0.122 (-0.238;0.007) | 0.49 |
|  | Others | -0.022 (-0.096 0.052) |  | -0.034 (-0.139 0.070) |  |
| **Intervention duration** | ≤Median | 0.029 (-0.059 0.117) | 0.21 | -0.036 (-0.175 0.102) | 0.71 |
|  | >Median | -0.065 (-0.165 0.034) |  | -0.054 (-0.152 0.043) |  |
| **Location** | North America | -0.044 (-0.141 0.053) | 0.69 | ;0.221 (-0.421;0.021) | 0.74 |
|  | Europe | 0.031 (-0.152 0.213) |  | -0.025 (-0.218 0.168) |  |
|  | South America | n. a. |  | -0.009 (-0.101 0.083) |  |
|  | Other | -0.015 (-0.087 0.058) |  | -0.007 (-0.242 0.227) |  |
| **Participants** | ≤Median | 0.024 (-0.133 0.182) | 0.41 | -0.024 (-0.158 0.110) | 0.66 |
|  | >Median | -0.044 (-0.125 0.036) |  | -0.069 (-0.208 0.069) |  |

Note: OSIs: oat supplementation intervention; DR: dietary restriction; n.a.: not available; WMD: Weighted mean difference.

# Leave one out sensitivity analysis

## Supplemental table 3. Leave one out sensitivity analysis for total cholesterol, in RCTs comparing OSIs vs. no OSI controls

| **Study omitted** | **Estimate** | **95% Confidence Interval** | |
| --- | --- | --- | --- |
| Adamsson et al. (2015) | -0.441 | -0.642 | -0.240 |
| Amundsen ÅL et al. (2003) | -0.416 | -0.621 | -0.211 |
| Biörklund et al. (2008) | -0.445 | -0.648 | -0.241 |
| Chang et al. (2013) | -0.416 | -0.617 | -0.214 |
| Connolly et al. (2016) | -0.337 | -0.45 | -0.223 |
| Ferguson et al., (2019) | -0.413 | -0.618 | -0.209 |
| Ferguson et al. (2019) | -0.379 | -0.578 | -0.179 |
| Gulati et al. (2017) | -0.429 | -0.665 | -0.194 |
| Ibrugger et al. (2013) | -0.427 | -0.630 | -0.224 |
| Kirby et al. (1981) | -0.398 | -0.594 | -0.201 |
| Van Horn L et al. (1991) | -0.426 | -0.651 | -0.201 |
| Robitaille et al. (2005) | -0.455 | -0.653 | -0.257 |
| Liatis et al. (2009) | -0.398 | -0.597 | -0.199 |
|  |  |  |  |
| **Combined** | **-0.415** | **-0.607** | **-0.223** |

## Supplemental table 4. Leave one out sensitivity analysis for HDL, in RCTs comparing OSIs vs. no OSIs controls

| **Study omitted** | **Estimate** | **95% Confidence Interval** | |
| --- | --- | --- | --- |
| Adamsson et al. (2015) | -0.010 | -0.039 | 0.018 |
| Amundsen ÅL et al. (2003) | -0.013 | -0.040 | 0.015 |
| Biörklund et al. (2008) | -0.014 | -0.043 | 0.014 |
| Chang et al. (2013) | -0.014 | -0.043 | 0.014 |
| Connolly et al. (2016) | -0.022 | -0.049 | 0.004 |
| Ferguson et al. (2019) | -0.014 | -0.042 | 0.013 |
| Ferguson et al. (2019) | -0.014 | -0.042 | 0.013 |
| Gulati et al. (2017) | -0.018 | -0.049 | 0.014 |
| Ibrugger et al. (2013) | -0.009 | -0.035 | 0.018 |
| Liatis et al. (2009) | -0.014 | -0.042 | 0.013 |
| Kirby et al. (1981) | -0.017 | -0.043 | 0.010 |
| Van Horn L et al. (1991) | -0.006 | -0.031 | 0.020 |
| Robitaille et al. (2005) | -0.022 | -0.046 | 0.003 |
|  |  |  |  |
| **Combined** | **-0.015** | **-0.041** | **0.012** |

## Supplemental table 5. Leave one out sensitivity analysis for LDL, in RCTs comparing OSIs vs. no OSIs controls

| **Study omitted** | **Estimate** | **95% Confidence Interval** | |
| --- | --- | --- | --- |
| Adamsson et al. (2015) | -0.304 | -0.392 | -0.215 |
| Amundsen ÅL et al. (2003) | -0.276 | -0.367 | -0.184 |
| Biörklund et al. (2008) | -0.308 | -0.394 | -0.223 |
| Chang et al. (2013) | -0.284 | -0.374 | -0.193 |
| Connolly et al. (2016) | -0.280 | -0.367 | -0.192 |
| Ferguson et al. (2019) | -0.278 | -0.369 | -0.186 |
| Ferguson et al. (2019) | -0.249 | -0.322 | -0.176 |
| Gulati et al. (2017) | -0.288 | -0.394 | -0.183 |
| Ibrugger et al. (2013) | -0.294 | -0.386 | -0.202 |
| Liatis et al. (2009) | -0.276 | -0.363 | -0.189 |
| Kirby et al. (1981) | -0.286 | -0.373 | -0.199 |
| Van Horn L et al. (1991) | -0.294 | -0.397 | -0.191 |
| Robitaille et al. (2005) | -0.306 | -0.393 | -0.219 |
|  |  |  |  |
| **Combined** | **-0.286** | **-0.372** | **-0.200** |

## Supplemental table 6. Leave one out sensitivity analysis for triglycerides comparing OSIs vs. no OSIs controls

| **Study omitted** | **Estimate** | **95% Confidence Interval** | |
| --- | --- | --- | --- |
| Kirby et al. (1981) | -0.021 | -0.099 | 0.057 |
| Adamsson et al. (2015) | -0.025 | -0.104 | 0.055 |
| Amundsen ÅL et al. (2003) | -0.041 | -0.108 | 0.026 |
| Biörklund et al. (2008) | -0.038 | -0.114 | 0.039 |
| Chang et al. (2013) | -0.014 | -0.089 | 0.061 |
| Connolly et al. (2016) | 0.002 | -0.064 | 0.068 |
| Gulati et al. (2017) | -0.021 | -0.112 | 0.069 |
| Van Horn L et al. (1991) | -0.008 | -0.098 | 0.082 |
| Liatis et al. (2009) | -0.017 | -0.094 | 0.059 |
| Robitaille et al. (2005) | -0.033 | -0.111 | 0.046 |
|  |  |  |  |
| **Combined** | **-0.022** | **-0.096** | **0.052** |

## Supplemental table 7. Leave one out sensitivity analysis for total cholesterol, in RCTs comparing OSIs+DR vs. DR alone

| **Study omitted** | **Estimate** | **95% Confidence Interval** | |
| --- | --- | --- | --- |
| Beck et al. (2010) | -0.433 | -0.563 | -0.303 |
| Beck et al. (2010) | -0.446 | -0.576 | -0.317 |
| Gerhardt et al. (1998) | -0.348 | -0.443 | -0.253 |
| Guevara-Cruz et al. (2012) | -0.450 | -0.593 | -0.307 |
| Kabir et al. (2002) | -0.422 | -0.555 | -0.289 |
| Li et al. (2016) | -0.466 | -0.621 | -0.311 |
| Li et al. (2016) | -0.447 | -0.623 | -0.271 |
| Reyna-Villasmil et al. (2007) | -0.465 | -0.604 | -0.325 |
| Saltzman et al. (2001) | -0.421 | -0.553 | -0.290 |
| Berg et al. (2003) | -0.390 | -0.514 | -0.266 |
|  |  |  |  |
| **Combined** | **-0.430** | **-0.556** | **-0.304** |

## Supplemental table 8. Leave one out sensitivity analysis for HDL, in RCTs comparing OSIs+DR vs. DR alone

| **Study omitted** | **Estimate** | **95% Confidence Interval** | |
| --- | --- | --- | --- |
| Berg et al. (2003) | -0.054 | -0.104 | -0.004 |
| Guevara-Cruz et al. (2012) | -0.062 | -0.115 | -0.008 |
| Leão et al. (2019) | -0.056 | -0.109 | -0.002 |
| Saltzman et al. (2001) | -0.069 | -0.118 | -0.020 |
| Beck et al. (2010) | -0.022 | -0.067 | 0.023 |
| Beck et al. (2010) | -0.026 | -0.071 | 0.020 |
| Gerhardt et al. (1998) | -0.054 | -0.104 | -0.005 |
| Kristensen et al. (2011) | -0.053 | -0.102 | -0.003 |
| Li et al. (2016) | -0.067 | -0.126 | -0.009 |
| Li et al. (2016) | -0.065 | -0.124 | -0.005 |
| Schweinlin et al. (2018) | -0.046 | -0.095 | 0.003 |
| Reyna-Villasmil et al. (2007) | -0.081 | -0.125 | -0.037 |
|  |  |  |  |
| **Combined** | **-0.054** | **-0.101** | **-0.006** |

## Supplemental table 9. Leave one out sensitivity analysis for LDL, in RCTs comparing OSIs+DR vs. DR alone

| **Study omitted** | **Estimate** | **95% Confidence Interval** | |
| --- | --- | --- | --- |
| Berg et al. (2003) | -0.219 | -0.340 | -0.097 |
| Guevara-Cruz et al. (2012) | -0.292 | -0.422 | -0.162 |
| Saltzman et al. (2001) | -0.247 | -0.375 | -0.120 |
| Beck et al. (2010) | -0.271 | -0.397 | -0.144 |
| Beck et al. (2010) | -0.280 | -0.406 | -0.154 |
| Gerhardt et al. (1998) | -0.215 | -0.330 | -0.099 |
| Kristensen et al. (2011) | -0.276 | -0.403 | -0.148 |
| Li et al. (2016) | -0.280 | -0.431 | -0.129 |
| Li et al. (2016) | -0.260 | -0.429 | -0.090 |
| Schweinlin et al. (2018) | -0.287 | -0.415 | -0.158 |
| Reyna-Villasmil et al. (2007) | -0.228 | -0.347 | -0.108 |
|  |  |  |  |
| **Combined** | **-0.26** | **-0.381** | **-0.138** |

## Supplemental table 10. Leave one out sensitivity analysis for triglycerides, in RCTs comparing OSIs+DR vs. DR alone

| **Study omitted** | **Estimate** | **95% Confidence Interval** | |
| --- | --- | --- | --- |
| Beck et al. (2010) | -0.045 | -0.144 | 0.054 |
| Beck et al. (2010) | -0.051 | -0.148 | 0.045 |
| Berg et al. (2003) | -0.044 | -0.139 | 0.051 |
| Gerhardt et al. (1998) | -0.029 | -0.125 | 0.066 |
| Guevara-Cruz et al. (2012) | -0.037 | -0.134 | 0.061 |
| Kabir et al. (2002) | -0.074 | -0.168 | 0.020 |
| Kristensen et al. (2011) | -0.042 | -0.142 | 0.057 |
| Leão et al. (2019) | -0.054 | -0.163 | 0.055 |
| Li et al. (2016) | -0.068 | -0.148 | 0.013 |
| Li et al. (2016) | -0.030 | -0.118 | 0.057 |
| Saltzman et al. (2001) | -0.040 | -0.139 | 0.058 |
| Schweinlin et al. (2018) | -0.041 | -0.138 | 0.056 |
| Uusitupa et al. (1992) | -0.044 | -0.143 | 0.055 |
| Reyna-Villasmil et al. (2007) | -0.059 | -0.163 | 0.045 |
|  |  |  |  |
| **Combined** | -0.047 | -0.141 | 0.046 |

## Supplemental table 11. Leave one out sensitivity analysis for glucose, in RCTs comparing OSIs+DR vs. DR alone

| **Study omitted** | **Estimate** | **95% Confidence Interval** | |
| --- | --- | --- | --- |
| Beck et al. (2010) | 0.020 | -0.169 | 0.209 |
| Beck et al. (2010) | 0.008 | -0.179 | 0.195 |
| Guevara-Cruz et al. (2012) | 0.034 | -0.207 | 0.274 |
| Kabir et al. (2002) | 0.018 | -0.163 | 0.200 |
| Leão et al. (2019) | -0.023 | -0.199 | 0.154 |
| Li et al. (2016) | 0.004 | -0.192 | 0.201 |
| Li et al. (2016) | 0.066 | -0.121 | 0.254 |
| Reyna-Villasmil et al. (2007) | 0.076 | -0.080 | 0.233 |
| Saltzman et al. (2001) | 0.041 | -0.144 | 0.226 |
| Schweinlin et al. (2018) | -0.024 | -0.203 | 0.154 |
|  |  |  |  |
| **Combined** | **0.021** | **-0.155** | **0.198** |

# Linear meta-regression analysis

## Supplemental figure 1 (a-d). Linear meta-regression analysis for total cholesterol for RCTs comparing OSIs vs. no OSIs controls

Bubble plots with fitted meta-regression line. The circles are sized according to the precision of each estimate with larger bubbles for more precise estimates.

## Supplemental figure 2 (a-d). Linear meta-regression analysis for HDL cholesterol for RCTs comparing OSIs vs no OSIs controls

Bubble plots with fitted meta-regression line. The circles are sized according to the precision of each estimate with larger bubbles for more precise estimates.

## Supplemental figure 3 (a-d). Linear meta-regression analysis for LDL cholesterol for RCTs comparing OSIs vs no OSIs controls

Bubble plots with fitted meta-regression line. The circles are sized according to the precision of each estimate with larger bubbles for more precise estimates.

## Supplemental figure 4 (a-d). Linear meta-regression analysis for triglycerides for RCTs comparing OSIs vs no OSIs controls

Bubble plots with fitted meta-regression line. The circles are sized according to the precision of each estimate with larger bubbles for more precise estimates.

## Supplemental figure 5 (a-d). Linear meta-regression analysis for total cholesterol for RCTs comparing OSIs+DR vs. DR alone

Bubble plots with fitted meta-regression line. The circles are sized according to the precision of each estimate with larger bubbles for more precise estimates.

## Supplemental figure 6 (a-d). Linear meta-regression analysis for HDL cholesterol for RCTs comparing OSIs+DR vs. DR alone

Bubble plots with fitted meta-regression line. The circles are sized according to the precision of each estimate with larger bubbles for more precise estimates.

## Supplemental figure 7 (a-d). Linear meta-regression analysis for LDL cholesterol for RCTs comparing OSIs+DR vs. DR alone

Bubble plots with fitted meta-regression line. The circles are sized according to the precision of each estimate with larger bubbles for more precise estimates.

## Supplemental figure 8 (a-d). Linear meta-regression analysis for triglycerides for RCTs comparing OSIs+DR vs. DR alone

Bubble plots with fitted meta-regression line. The circles are sized according to the precision of each estimate with larger bubbles for more precise estimates.

# Publication bias analysis

## Supplemental figure 9. Publication bias for RCTs comparing OSIs vs. no OSIs controls for total cholesterol

## Supplemental figure 10. Publication bias for RCTs comparing OSIs vs. no OSIs controls for HDL cholesterol

## Supplemental figure 11. Publication bias for RCTs comparing OSIs vs. no OSIs controls for LDL cholesterol

## Supplemental figure 12. Publication bias for RCTs comparing OSIs vs. no OSIs controls for triglycerides

## Supplemental figure 13. Publication bias for RCTs comparing OSIs vs. no OSIs controls for body mass index

## Supplemental figure 14. Publication bias for RCTs comparing OSIs vs. no OSIs controls for body weight

## Supplemental figure 15. Publication bias for RCTs comparing OSIs vs. no OSIs controls for systolic blood pressure

## Supplemental figure 16. Publication bias for RCTs comparing OSIs vs. no OSIs controls for diastolic blood pressure

## Supplemental figure 17. Publication bias for RCTs comparing OSIs+DR vs. DR alone, for body mass index

## Supplemental figure 18. Publication bias for RCTs comparing OSIs+DR vs. DR alone, for total body weight

## Supplemental figure 19. Publication bias for RCTs comparing OSIs+DR vs. DR alone, for total waist circumference

## Supplemental figure 20. Publication bias for RCTs comparing OSIs+DR vs. DR alone, for total glucose

## Supplemental figure 21. Publication bias for RCTs comparing OSIs+DR vs. DR alone, for total systolic blood pressure

## Supplemental figure 22. Publication bias for RCTs comparing OSIs+DR vs. DR alone, for diastolic blood pressure
